# Identification of DNA methylation patterns and biomarkers for clear-cell renal cell carcinoma by multi-omics data analysis

**DOI:** 10.7717/peerj.9654

**Published:** 2020-08-03

**Authors:** Pengfei Liu, Weidong Tian

**Affiliations:** 1 State Key Laboratory of Genetic Engineering and Collaborative Innovation Center for Genetics and Development, School of Life Sciences, Fudan University, Shanghai, China; 2 Department of Biostatistics and Computational Biology, School of Life Sciences, Fudan University, Shanghai, China; 3 Children’s Hospital of Fudan University, Shanghai, China

**Keywords:** Multi-omics, DNA methylation (DNAm), Clear cell renal cell carcinoma (ccRCC), Diagnostic biomarkers, Prognostic biomarkers

## Abstract

**Background:**

Tumorigenesis is highly heterogeneous, and using clinicopathological signatures only is not enough to effectively distinguish clear cell renal cell carcinoma (ccRCC) and improve risk stratification of patients. DNA methylation (DNAm) with the stability and reversibility often occurs in the early stage of tumorigenesis. Disorders of transcription and metabolism are also an important molecular mechanisms of tumorigenesis. Therefore, it is necessary to identify effective biomarkers involved in tumorigenesis through multi-omics analysis, and these biomarkers also provide new potential therapeutic targets.

**Method:**

The discovery stage involved 160 pairs of ccRCC and matched normal tissues for investigation of DNAm and biomarkers as well as 318 cases of ccRCC including clinical signatures. Correlation analysis of epigenetic, transcriptomic and metabolomic data revealed the connection and discordance among multi-omics and the deregulated functional modules. Diagnostic or prognostic biomarkers were obtained by the correlation analysis, the Least Absolute Shrinkage and Selection Operator (LASSO) and the LASSO-Cox methods. Two classifiers were established based on random forest (RF) and LASSO-Cox algorithms in training datasets. Seven independent datasets were used to evaluate robustness and universality. The molecular biological function of biomarkers were investigated using DAVID and *GeneMANIA*.

**Results:**

Based on multi-omics analysis, the epigenetic measurements uniquely identified DNAm dysregulation of cellular mechanisms resulting in transcriptomic alterations, including cell proliferation, immune response and inflammation. Combination of the gene co-expression network and metabolic network identified 134 CpG sites (CpGs) as potential biomarkers. Based on the LASSO and RF algorithms, five CpGs were obtained to build a diagnostic classifierwith better classification performance (AUC > 99%). A eight-CpG-based prognostic classifier was obtained to improve risk stratification (hazard ratio (HR) > 4; log-rank test, *p*-value < 0.01). Based on independent datasets and seven additional cancers, the diagnostic and prognostic classifiers also had better robustness and stability. The molecular biological function of genes with abnormal methylation were significantly associated with glycolysis/gluconeogenesis and signal transduction.

**Conclusion:**

The present study provides a comprehensive analysis of ccRCC using multi-omics data. These findings indicated that multi-omics analysis could identify some novel epigenetic factors, which were the most important causes of advanced cancer and poor clinical prognosis. Diagnostic and prognostic biomarkers were identified, which provided a promising avenue to develop effective therapies for ccRCC.

## Introduction

According to the latest global cancer statistics in 2018, renal cell carcinoma (RCC) is among the top ten most common malignancies in which the incidence and mortality accounts for more than 3% of all human malignancies (Bray et al., 2018). Among all types of RCC, the major histological subtype is clear cell renal cell carcinoma (ccRCC), which has the worst prognosis and more complex heterogeneity, accounting for 80–90% of all RCC cases (Hsieh et al., 2017). To understand the potential molecular alterations that drive ccRCC oncogenesis, The Cancer Genome Atlas (TCGA) project based on single-omics analysis from different working groups has emphasized the importance of molecular characterization and histological assessment to stratify ccRCC (Linehan & Ricketts, 2019; Prakoura & Chatziantoniou, 2017; Sigdel et al., 2019). These results indicate the complexity of ccRCC tumorigenesis and suggest that single-omics insufficient to fully investigate this cancer type to identify effective diagnostic or prognostic biomarkers. Furthermore, it is difficult for pathologists to distinguish ccRCC based on morphology and immunohistochemistry (Bucur & Zhao, 2018). Therefore, it is necessary to identify potential diagnostic or prognostic molecular biomarkers for ccRCC (Clark et al., 2020).

In the early stage, the diagnosis and prognosis of ccRCC was mainly based on abdominal imaging and clinicopathologic signatures, and those strategies have some contingency with an approximate accuracy of 80% (Lightfoot et al., 2000). The diagnostic and prognostic biomarkers were also identified at different omics levels, including microRNA (Guan et al., 2018), long noncoding RNA (Qu et al., 2018), mRNA (Chen et al., 2019; Wang et al., 2017) and proteins (Lopez et al., 2016). However, those biomarkers have some obvious drawbacks, including unstable molecular structure, the fact that they are easily affected by external factors, lower abundance and difficulty in being detected, thus limiting their clinical application. The epigenetic changes of cancer cells have been proved to be one of the important mechanisms of tumorigenesis, in which the abnormal methylation of gene promoter regions, especially CpG island regions, is also known to be an important factors that caused gene suppression (Bibikova et al., 2011). The TCGA project and other studies have shown that DNAm can be used as the cancer-specific characteristic for the development of diagnostic or prognostic biomarkers and targeted therapy (Linehan & Ricketts, 2019). For example, Skrypkina et al. (2016) reported that the sensitivity of a model based on an individual gene ranges from 51.9% to 62.9%. At the same time, the use of a combination of two or three genes led to a significant increase in sensitivity (77.9–92.3%) and specificity (86.7–93.3%). However, the researches were only based on the epigenetic level, and it was not sufficient to assess the effect of aberrant methylation status on cancer progression. Based on high-throughput technology, the previous studies shown that many significantly different epigenetic factors did not always cause abnormality in gene expression. The abnormal gene expression is also one of the important factors that cause cancer or disease (Cui et al., 2016; Linehan & Ricketts, 2019; Zhao et al., 2020). Meanwhile, Xu et al. (2020) developed a seven-CpG-based classifier for ccRCC prognosis by integrating DNA methylation (DNAm) and gene expression. However, owing to significant reprograming of metabolic pathways, the integration analysis based on DNAm and gene expression may be not detailed enough to identify aberrant patterns of expression changes in many biological pathways (Furuta et al., 2010). As is known to all, the metabolic reprograming of cancer cells significantly influences some important physiological and biochemical reactions, including cell invasion, adhesion and immune response. Although not the case for all cancers, RCC has been regarded as a cancer driven by metabolic disorders due to the abnormal expression of genes that regulate various metabolic pathways, such as *FH* and *SDHB* in the Krebs cycle (Linehan, Srinivasan & Schmidt, 2010). Hence, the analysis of the expression patterns of enzyme-encoding genes in metabolic pathways was added to our research, which will complement our understanding of the pathogenesis and aid in providing new potential biomarkers or targets involved in tumorigenesis.

In this study, the multi-omics analysis of epigenetic measurements uniquely identified DNAm dysregulation of cellular mechanisms resulting in transcriptomic alterations, including cell proliferation, immune response and inflammation. Combination of the gene co-expression network and metabolic network, a five-CpG-based classifier with better classification performance was obtained using Least Absolute Shrinkage and Selection Operator (LASSO) and RF algorithm. An eight-CpG-based prognostic classifier was developed to identify the high-risk groups more accurately (hazard ratio (HR) > 4; log-rank test, *p*-value < 0.01). Based on independent datasets and seven additional cancers, the diagnostic and prognostic classifiers also had better robustness and stability. The molecular function analysis of biomarkers with abnormal methylation indicated that they were significantly related to glycolysis/gluconeogenesis and signal transduction. Thus, our studies not only identified biomarkers involved in important metabolic pathways in tumorigenesis, but also provided novel therapeutic targets for treating advanced RCC in a general manner.

## Materials and Methods

### Workflow chart and samples preparation

**Figure 1:**
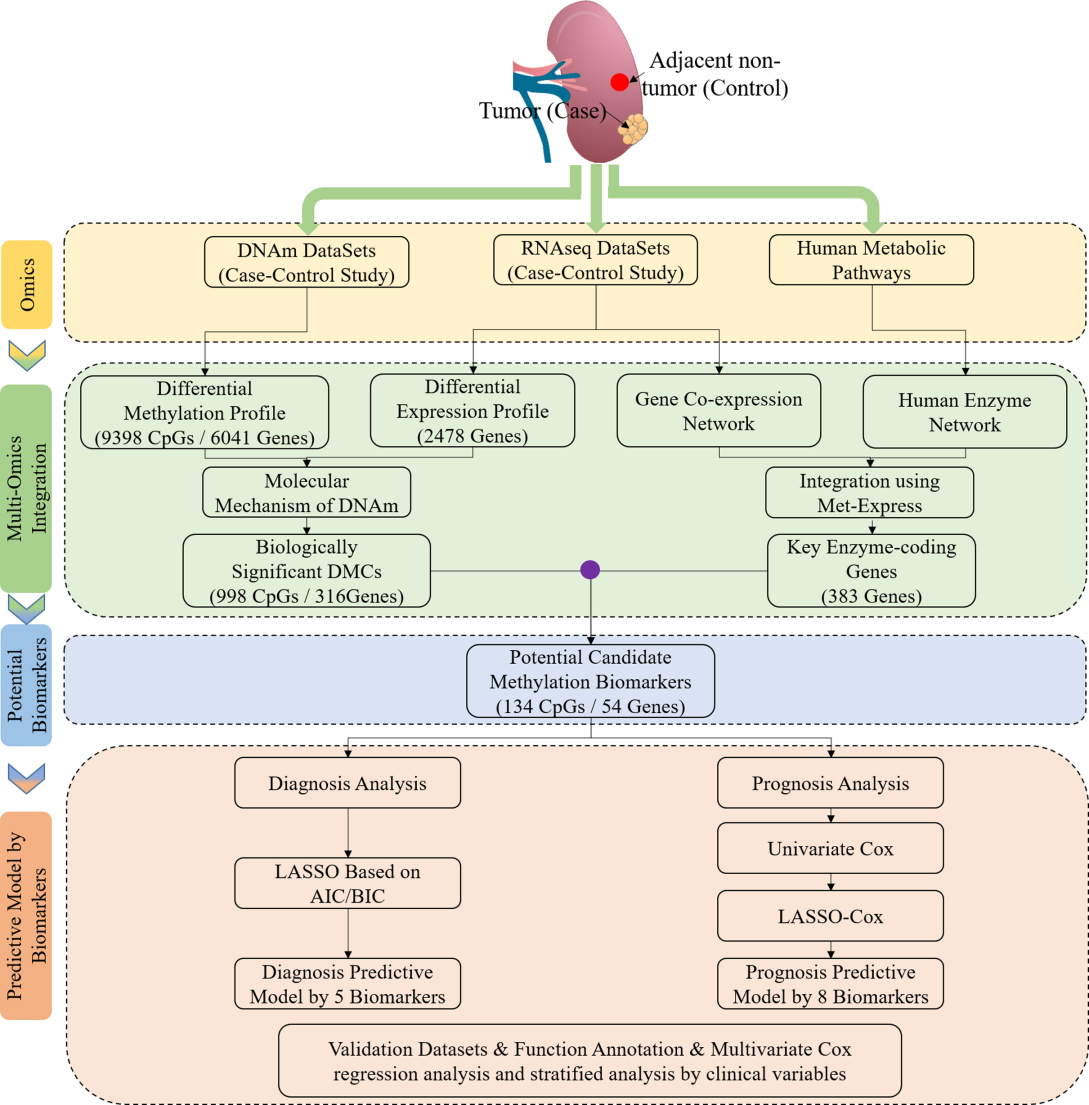
Study flowchart of data generation and analysis. DMC, differentially methylated CpG sites; DNAm, DNA methylation. Integrated methylation signatures on ccRCC and non-tumor tissues were used to identify 134 candidate biomarkers. Diagnostic biomarker selection: LASSO was applied to a training cohort to identify a final selection of five biomarkers. These five markers were applied to a validation cohort. Prognostic biomarker selection: univariant-cox and LASSO-Cox were applied to a training cohort with survival data to identify a final selection of eight biomarkers. These eight biomarkers were applied to a validation cohort with survival data.

Figure 1 shows the study workflow chart. In this study, DNAm (290 tumor and 160 normal), mRNA (526 tumor and 72 normal) and clinical data (318 cases of ccRCC) were collected from TCGA (https://portal.gdc.cancer.gov/). Of note, 160 cases of ccRCC had both ccRCC tissues and matched adjacent normal tissues in terms of DNAm profiles. In addition, 24 cases of ccRCC, which had both ccRCC tissues and matched adjacent normal tissues in terms of mRNA profiles, had both DNAm and mRNA expression profiles. The human KEGG Markup Language (KGML) files were obtained from the KEGG database (http://www.genome.jp/kegg/), and these files were used to predict key enzyme-coding genes. In addition, the DNAm status for the following five additional cancers were derived from TCGA, including both DNAm and gene expression profiles: BRCA (774 tumor and 82 normal), COAD (292 tumor and 38 normal), LIHC (380 tumor and 50 normal), LUAD (455 tumor and 32 normal) and PRAD (502 tumor, 50 normal). Moreover, the following two cancers were obtained from TCGA, including only DNAm or gene expression profiles: KIRP (274 tumor and 45 normal) and UCEC (425 tumor and 34 normal).

### Differential analysis of mRNA expression and DNAm

Based on DNAm profiles, probes with more than 50% missing values were deleted. The “impute.knn” function (R version 3.5.0) was used to impute the remaining missing values. CpG probes in chromosomes X and Y were excluded, and CpG probes identified as non-unique in the genome as well as CpG probes known as single-nucleotide polymorphisms were also excluded (Noushmehr et al., 2010; Selamat et al., 2012). Differentially methylated CpG sites (CpGs) were detected using the non-parametric testing method (Selamat et al., 2012), and *p*-values were adjusted using the false discovery rate (FDR) method in R (R version 3.5.0; p.adjust (method = “BH”)). CpG probes were filtered as described below, and these filtered CpG probes were considered to be statistically significantly differentially methylated. FDR-values were less than 0.05, and the absolute values of the mean β-value difference between ccRCC and non-ccRCC tissues were greater than 0.2 (Selamat et al., 2012). In addition, the differentially expressed genes were detected based on “limma” function in Bioconductor 3.10. The significantly differentially expressed genes were identified using the following cutoffs: FDR-value < 0.05 and |log2 fold change| > 1.

Finally, we investigated the correlation between DNAm and mRNA expression. Pearson correlation coefficients for each gene or CpG were calculated between mRNA expression and DNAm using the Spearman’s rank correlation coefficient. The correlation was considered statistically significant when the absolute value of Spearman’s Rho was greater than 0.2 and the FDR-values were less than 0.05 (Cancer Genome Atlas Research, 2011; Cheng et al., 2018).

### Screening key enzyme-coding genes by met-express

In previous study, the gene co-expression and genome-scale metabolic network were integrated to identify the key enzyme-coding genes in human diseases. Here, we briefly describe the algorithm (Chen et al., 2013). (1) Based on transcriptome data, the Pearson correlation coefficients (PCC) were calculated for all pairs of enzyme-coding genes. The top three associated genes with the highest PCC for each gene were used to construct gene co-expression network. Qcut (Ruan & Zhang, 2008) was used to divide the network into gene co-expression modules, and we only remain some modules including more than 10 genes. (2) We carried out the reconfiguration of metabolic network based on KGML files (http://www.genome.jp/kegg/) containing the information of critical metabolic reactions, key enzyme-coding genes and corresponding metabolites. (3) The importance score was calculated for each gene by integrating with gene cox-expression network and reconstructed metabolic network. Then, the median importance score was used for a threshold to select the candidate key enzyme-coding genes.

### Identification of candidate tissue-specific diagnostic biomarkers

For 24 patients who have both mRNA expression and DNAm profiles, first-level feature selection was performed though the following steps. First, DNAm profiles were obtained by Wilcoxon rank-sum test to select differentially methylated CpGs (FDR < 0.05, |Δβ_mean_| > 0.2); mRNA expression profiles were obtained by “limma” function (R version 3.5.0) to select differentially expressed genes (FDR < 0.05, |log2 fold change| > 1). Second, the Rho was calculated between DNAm and mRNA expression by Spearman’s rank correlation coefficient to select genes with higher correlation coefficient (FDR < 0.05, |Rho| > 0.2). For genes with multiple CpGs measuring Rho, we selected the CpG with the highest absolute value of Rho. Third, the key enzyme-coding genes in cancers were selected by Met-Express (Chen et al., 2013). Finally, CpGs obtained from the above three steps were intersected to obtain the candidate CpGs, namely the candidate tissue-specific diagnostic biomarkers for first-level feature selection.

To build the effective diagnostic model, it was essential to identify a small set of biomarkers. Thus, some redundant biomarkers were excluded to avoid over-fitting and achieve the best prediction performance (Tang et al., 2018). After the first-level feature selection, the number of those CpGs was still large and redundant. Therefore, we performed a second-level feature selection by the LASSO method (Tibshirani, 1996) to further remove redundant CpGs and identify tissue-specific diagnostic biomarkers.

### Evaluation and modeling of candidate tissue-specific diagnostic biomarkers

In this study, the random forest (RF) classifier was built based on the tissue-specific diagnostic biomarkers to evaluate performance of those biomarkers. Apart from 24 patients for the variable selection, 136 patients who have both tumor and adjacent normal tissue were randomly allocated to the training and testing sets. The training set containing 90 patients was used to optimize the parameters of classifiers and build classifiers. The testing set containing 46 patients was used to evaluate performance of the classifiers. Ten-fold cross-validation was used during the training and testing of classifiers, and the performance of classifier was evaluated based on area under the ROC Curve (AUC). Meanwhile, we also introduced the sensitivity and specificity to assist in evaluating the performance of the diagnostic classifier.

### Validation based on independent data sets and literatures

In this study, the Illumina HumanMethylation450 BeadChip data was comprehensive queried by using keywords focused on “renal clear cell carcinoma”, “ccRCC” and “methylation”. In the process of data retrieval, the following conditions should be met as much as possible which included the initial experimental research providing a comparison of ccRCC and non-ccRCC tissues, the patients without receive neoadjuvant therapy. We obtained two independent datasets, including GSE70303 (Becket et al., 2016) (46 tumor and non-tumor tissues from GEO database) and E-MTAB-2007 (Sato et al., 2013) (106 tumor and non-tumor tissues from ArrayExpress database), to perform verification of candidate tissue-specific biomarkers.

### Construction of prognostic model

Apart from 24 patients for the variable selection, we split the rest of ccRCC patients in a training dataset and a validation dataset and explored to build a predictive model for prognostic and survival analysis. We used 192 cases for training and 98 cases for validation. We applied a sequential model-based variable selection to screen markers for predicting survival outcome (Ding, Chen & Shi, 2019; Xu et al., 2017). Based on the candidate biomarkers, we first fitted a univariate Cox proportional hazards model by using each marker as the covariate. A marker with *p*-value < 0.05 from the Wald statistic was retained in the dataset. Second, we used a similar strategy in a prognosis marker selecting process based on the LASSO-Cox method to decrease the marker numbers to a reasonable range (less than events). The above analysis generated final markers with non-zero coefficient to construct a prognostic signature using R package “glmnet”. Based on the penalized Cox regression model, we obtained a combined prognostic score (designated as risk score) for each individual. The risk-score for patients is calculated as follows:

$${\rm Risk\; score\; for\; patients = }\mathop \sum \nolimits_{{ i = 1}}^{N} {\rm (cofficients\; of\; each\; CpGs\; \times \; \beta - values\; of\; each\; \; CpGs)}$$

To validate the predictive classifier, we calculated a risk-score for each patient in the validation dataset using the coefficient estimated from the training dataset. By grouping the risk-score based on its median, we formed high and low risk-score groups with roughly equal number of observations. The Kaplan–Meier estimator and log-rank test were performed to test whether the median survival time for two groups was significantly different. All analyses were conducted in R version 3.5.0 with the “glmnet” and “survival” packages.

### Functional enrichment analysis based on gene ontology

The CpG and gene annotations were downloaded from TCGA (http://tcga-data.nci.nih.gov/tcga/tcgaPlatformDesign.jsp) and GECODE (https://www.encodeproject.org/files/gencode.v22.annotation/). Genes with abnormal methylation were analyzed for Gene Ontology (GO) analysis or Kyoto Encyclopedia of Genes (KEGG) pathway enrichment analysis by using the web version of DAVID (Da Huang, Sherman & Lempicki, 2009). GO/KEGG-terms have an adjusted *p*-values which were less than 0.05. The genes were uploaded to the online database *GeneMANIA* (http://genemania.org; version 3.6) (Warde-Farley et al., 2010) to explore their interactions at the protein level with an interaction score > 0.4 as the cutoff value. Afterward, the network was visualized in software Cytoscape (version 3.6.1).

### Statistical analysis

Based on Euclidean distance matrix and complete-linkage method, unsupervised hierarchical clustering was carried out to verify the reliability of the analysis method about CpGs in R version 3.5.0 with the “heatmap” and “dist” functions. To discuss the widespread nature of cancer-specific methylation alterations and the impact of aberrant methylation on gene expression, the Chi-square test and Wilcoxon rank-sum test were used in R version 3.5.0 with the “chisq.test” and “wilcox.text” functions. When *p*-values were less than 0.05, it was deemed to be statistically significant. The diagnostic models were built in scikit-learn framework (version 0.20.3; Python 3.6..8) with the “sklearn.ensemble.RandomForestClassifier” algorithm. The parameters of models was tuned in scikit-learn framework (version 0.20.3; Python 3.6.8) with the “sklearn.grid_search.GridSearchCV” algorithm. The hyper-parameters included the number of trees (10 to 100), the criterion (“gini” and “entropy”), “oob_score” in the RF. The default values were used when other parameters were not provided in functions or classifier.

## Results

In our study, 160 ccRCC and matched normal tissues were obtained through the TCGA projects. Among 160 patients, the mean age was 63 years, and the ratios for the sex (female to male) and cancer staging (stage I–II to stage III–IV) were 1:2 and 7:9, respectively. Although smoking, obesity and hypertension could affect the risk of ccRCC, TCGA projects did not provide the clinical signatures (NULL). The study flowchart is shown in Fig. 1. The patients’ characteristics are presented in Table S1.

### Identification and landscape of differentially methylation in ccRCC

**Figure 2:**
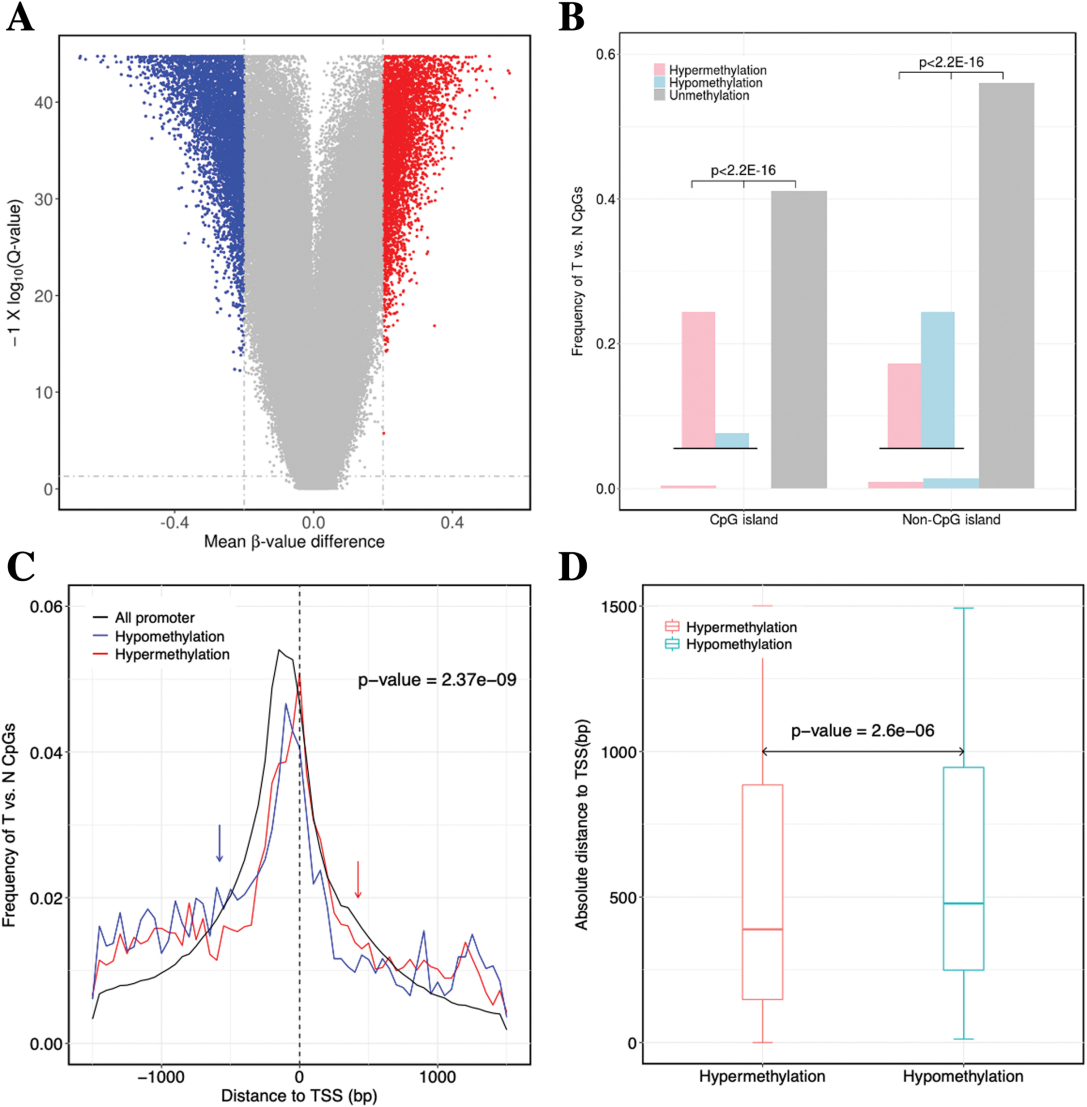
Identification of DNA methylation difference between ccRCC and matched adjacent non-ccRCC samples. (A) Volcano plot of the differential DNA methylation analysis (*X*-axis). Mean β-value difference (mean ccRCC–mean non-ccRCC); (*Y*-axis) *Q*-values for each CpG sites (−1 × log_10_ scale). Blue points represent hypomethylated CpG sites; red points represent hypermethylated. (B) Proportions of CpG sites on CGIs and non-CGIs. Red bar represents hypermethylated CpG sites; blue bar represents hypomethylated CpG sites; gray bar represents unmethylated CpG sites. *p* Values were computed with χ^2^ test. (C) Normalized histogram of CpG sites with respect to TSSs distance. Red line represents hypermethylated CpG sites; blue line represents hypomethylated CpG sites; black line represents background distribution of all promoter CpG sites. Red and blue arrows represent significantly difference between the characteristics of hyper-and hypomethylated CpG sites in promoters. Up-and downstream distances from TSSs are represented by positive and negative values, respectively. *p* Values were computed with the Wilcoxon rank sum test. (D) Box plot of TSS absolute distance for CpG sites. Red box represents the hypermethylated promoter CpG sites were located in CGI regions; blue box represents the hypomethylated promoter CpG sites were located in CGI regions.

Based on the differentially methylated CpGs, the exploratory hierarchical clustering was first performed (Fig. S1). The methylation profiles of ccRCC and non-ccRCC resulted in separate groups, suggesting a potential difference. In our study, we obtained 4367 CpGs (corresponding to 2,490 genes) that were significantly hypermethylated in ccRCC and 5,031 CpGs (corresponding to 3,551 genes) that were significantly hypomethylated (Fig. 2A). Some of the most hypermethylated loci contained *HOXA5*, involving 31 CpGs in the CGI (29/31) or N_Shore (2/31) and coding for a DNA-binding transcription factor, which regulated gene expressions, cell differentiation and morphogenesis (Shenoy et al., 2015). Some of the most hypomethylated loci contained *CXCL8*, which promoted angiogenesis and metastasis (Shenoy et al., 2015). To investigate the biological functions of genes with abnormal methylation, the target genes was conducted by DAVID (Da Huang, Sherman & Lempicki, 2009). Most genes were significantly enriched in the important biological processes, including cell adhesion, signal transduction and positive regulation of transcription (Benjamini–Hochberg (BH) adjusted *p*-value < 0.05) (Table 1), indicating that these genes played an important role in tumorigenesis.

**Table 1:** Top 10 Gene Ontology (GO) biological process (BP) based on genes with aberrant methylation.

GO	Term	Count	FDR
GO:0007155	Cell adhesion	198	6.87E−16
GO:0007165	Signal transduction	391	1.89E−10
GO:0045944	Positive regulation of transcription from RNA polymerase II promoter	339	2.41E−10
GO:0043547	Positive regulation of GTPase activity	210	1.12E−08
GO:0007156	Homophilic cell adhesion via plasma membrane adhesion molecules	79	1.36E−08
GO:0001525	Angiogenesis	96	2.43E−06
GO:0007399	Nervous system development	116	3.93E−06
GO:0035556	Intracellular signal transduction	149	2.50E−05
GO:0000122	Negative regulation of transcription from RNA polymerase II promoter	241	2.99E−05
GO:0060333	Interferon-gamma-mediated signaling pathway	38	7.91E−04

For the significantly differentially methylated CpGs, we also analyzed whether they are related with CGIs and whether they are located in the promoter or gene body (Fig. S2). We identified 9398 CpGs defined as being differentially hyper- or hypomethylated. Moreover, 33.33% and 31.96% of CpGs located in promoter regions, which was defined as 1.5 kb up-or downstream from the transcription start site (TSS), were significantly hyper-or hypomethylated, respectively. We found no statistically significant difference between the degree of methylation and whether the CpGs were located in the gene promoter regions (*p*-value = 0.17, Fisher’s exact test). However, 31.87% and 3.79% of CpGs located in CGIs were significantly hyper-or hypomethylated (*p*-value < 2.2 × 10^−16^, Fisher’s exact test), respectively. These results showed a significant difference between the methylation characteristics of CGIs and non-CGIs for differentially methylated CpGs. Based on CGI regions, the significantly hypermethylated CpGs were highly enriched in CGIs (*p*-value < 2.2 × 10^−16^, χ^2^ test), whereas hypomethylated CpGs were enriched in non-CGIs (*p*-value < 2.2 × 10^−16^, χ^2^ test) (Fig. 2B). Compared to hypomethylated CpGs, hypermethylated CpGs were located closer to TSSs (*p*-value = 2.37 × 10^−9^, Wilcoxon rank sum test) (Fig. 2C). Compared to CGIs containing hypomethylated CpGs, CGIs containing hypermethylated CpGs were located closer to TSSs (*p*-value = 2.6 × 10^−6^, Wilcoxon rank sum test) (Fig. 2D).

In conclusion, the hypermethylated CpGs were preferentially enriched in CGIs, but CpGs exhibiting abnormal hypomethylation in tumors were frequently occurred in non-CGI regions. Furthermore, the CGI and promoter regions containing hypermethylated CpGs were located closer to TSSs.

### Impact of the cancer-associated differentially methylation no gene expression

To identify the tumor-associated methylation alterations with concomitant changes at the transcriptional levels, the correlation analysis of the methylation and transcriptome data were performed. By focusing on CpGs and their nearest neighbor genes (called “*cis*-interactions”) that fall within 1,500 bp upstream of TSS to the end of gene body, genes with abnormal methylation were studied (Fig. S3). By comparing 1,154 significantly differentially methylated CpGs located in 316 associated genes, 998 (86.48%, 998 of 1,154 CpGs) CpGs with the significant correlations between DNAm and gene expression were identified.

**Figure 3:**
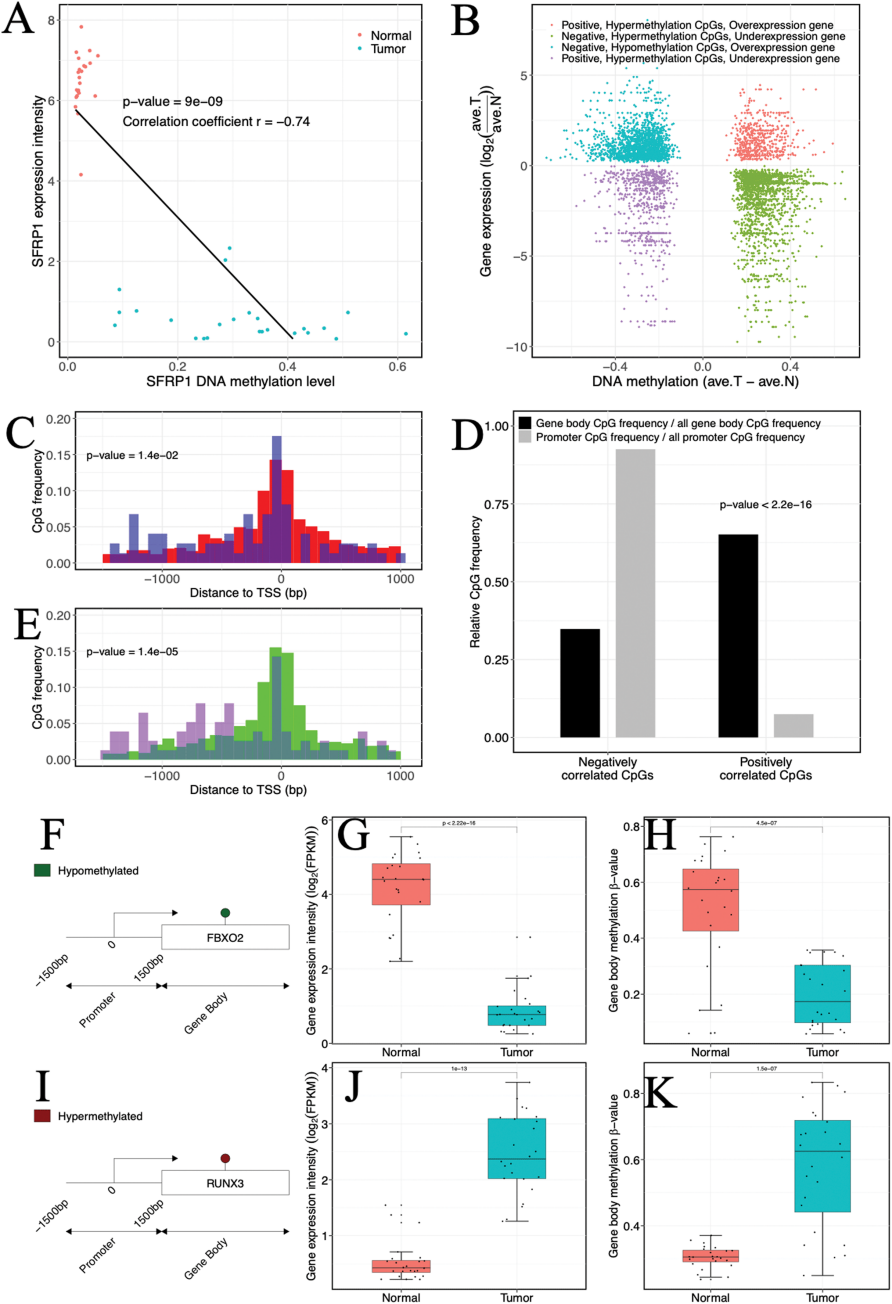
Impact of abnormal methylation levels on gene expression in ccRCC. (A) Example of a gene (SFRP1) showing a negative gene expression-DNA methylation relationship. Blue, ccRCC tumors; red, matched adjacent normal tissues. (B) Starburst plot of gene expression and DNA methylation differences in ccRCC and matched adjacent normal tissues. Only CpG sites (points) demonstrating significant DNA methylation-gene expression correlations are shown. *X* axis, differential DNA methylation levels between ccRCC and matched adjacent normal tissues. *Y* axis, differential gene expression levels between ccRCC and matched adjacent normal tissues. (C, E) Relationships of positively correlated CpG sites to TSSs. (C) Normalized histogram of positively correlated CpG sites hypermethylated and over-expressed (blue) compared to conventional negatively correlated CpG sites (red). (E) Histogram of positively correlated CpG sites hypomethylated and under-expressed (purple) compared to conventional negatively correlated CpG sites (green). (D) Bar graphs exhibiting ratios of gene body (black) and promoter (gray) CpG frequencies within negatively and positively correlated CpG sites to all gene body and promoter CpG site frequencies. *p* Values were computed with Chi-square test. (F, I) (Left) Schematic representation of genes including significantly positively correlated gene body CpG sites for FBXO2 (F) and RUNX3 (I). (G, J) Box plots comparing gene expression levels associated with positively correlated gene body CpG sites in ccRCC and matched adjacent normal tissue. (H, K) Box plots comparing gene body methylation levels of positively correlated gene body CpG sites in ccRCC and matched adjacent normal tissue.

In accordance with DNAm mechanism to silence or enhance local transcription, 68% of CpGs identified as significantly correlated were negative (681of 998 CpGs) (Figs. 3A and 3B). A portion of the CpGs were primarily in gene promoter regions (68.14%, 464 of 681 CpGs). Of these, 336 CpGs (72.41%, 336 of 464 CpGs) were statistically significantly hypermethylated and inhibited gene expression, while 128 CpGs (27.59%, 128 of 464 CpGs) were hypomethylated and caused over-expression of genes, indicating that abnormal DNAm might have functional consequence in approximately 40% (464 of 1,154 CpGs) of CpGs associated with genes. The hypermethylated and under-expressed genes included Secreted Fzd-related protein 1 (*SFRP1*), which was consistent with previous ccRCC studies (Awakura et al., 2008; Gumz et al., 2007), and *PIC3K2G*, which was a known cancer susceptibility gene (Brown et al., 2015) (Table S2). The WNT antagonist, *SFRP1*, was also a tumor suppressor that regulates cell proliferation and blood vessel formation (Atschekzei et al., 2012). CGI methylation located in *SFRP1* has also been shown to be indirectly associated with tumor progression and tumor stage (Urakami et al., 2006) as well as to directly lead to the occurrence of poor prognosis in RCC patients (Morris et al., 2010; Saini et al., 2009). *PIK3C2G* encoded a phosphoinositide three kinase (PI3K) subtype, which was involved in the regulation of signaling pathways, such as cell proliferation, oncogenic transformation, cell survival and cell migration (Brown et al., 2015; Freitag et al., 2015). Conversely, the hypomethylated and over-expressed genes included single transduction-related genes (*GUCA2B*) as well as functional genes involved in cell differentiation (*PPDPFL*), PPAR signaling pathway (*FABP6*), HIF-1-alpha transcription factor network (*CA9*), and receptor-associated protein activity (*SLC6A3*) (Table S2). The molecular biological functions uncovered that hypermethylated/under-expressed genes were associated with the immune response, inflammatory response and cell adhesion (Table S3). However, hypomethylated/over-expressed genes were associated with cell or tissue development, intracellular transport and cellular secretion (Table S3).

In our investigation, a certain proportion (31.76%, 317 of 998 CpGs) of positive correlation was observed (Fig. 3B). Of which a lower percentage (10.73%, 34 of 317 CpGs) of the positively correlated CpGs was located in promoter regions, but our analysis indicated that these CpGs might be related to atypical TSS distances compared to negatively correlated CpGs. Specifically, the hypermethylated and over-expressed CpGs tended to lie closer to TSSs (*p*-value = 1.4 × 10^−5^, Wilcoxon rank sum test), whereas the hypomethylated and under-expressed CpGs were located further upstream of TSSs compared to their cognate negatively correlated (hypomethylated and over-expressed) CpGs (*p*-value = 1.4 × 10^−2^, Wilcoxon rank sum test) (Figs. 3C and 3E). The molecular biological functions revealed that the hypermethylated CpGs with positive correlation were mainly enriched in immune processes and cell motility, whereas the hypomethylated CpGs with positive correlation were enriched in cell proliferation and development. Furthermore, CpGs that appeared to be positively correlated were inclined to be located in gene bodies (*p*-value < 2.2 × 10^−16^, χ^2^ test) (Fig. 3D). In the gene bodies, the hypermethylated and over-expressed genes included *RUNX3*, and hypomethylated and under-expressed genes included *FBXO2* (Figs. 3F–3K). Intriguingly, several genes showing positive correlation in gene bodies have been previously linked to cancer, including *MUC15* (Huang et al., 2009), *HEPACAM2* (Klopfleisch et al., 2010), *CA10* (Romeo et al., 2009), *NRG1* (Huang et al., 2004) and *RAB25* (Mitra, Cheng & Mills, 2012). In addition, ~26% of genes that exhibited positive correlation and were located in gene bodies also exhibited negative correlations in promoters (Table S4), including *RUNX3* and *TMEM30B* (Fig. S4). Based on four independent datasets including two methylation datasets (GSE70303 and GSE61441) and two gene expression profiles (GSE40435 and GSE76351), we also found the same events (Table S5). These results suggested that epigenetic alterations had a dual effect on gene expression, that is, genes with hypermethylation in the promoter and hypomethylation in the gene body were suppressed or genes with hypomethylation in the promoter and hypermethylation in the gene body were activated. The occurrence of the “tandem control” mechanisms indicated that methylation alterations located in the promoter might interact with methylation status in the gene body, thereby regulating the expression of cancer-associated genes.

### Identification of tissue-specific biomarkers based on multi-omics integration

**Figure 4:**
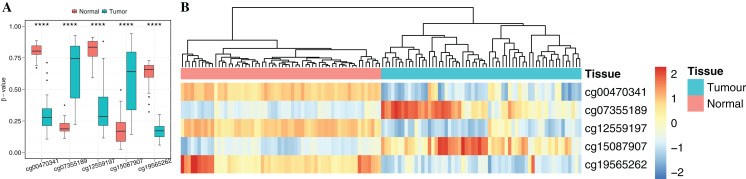
Identification of the DNA methylation-based diagnostic biomarkers. (A) The methylation values and standard deviations of five diagnostic biomarkers were from the benign-adjacent and patient cancer tissues. The *p*-value was calculated through Wilcoxon rank sum test, and “****” means *p*-value was less than 0.001. (B) Unsupervised hierarchical clustering of five methylation biomarkers selected for use in the diagnostic prediction model in the independent dataset, in which the metric of similarity was Pearson’s correlation based on the methylation levels.

To obtain cancer tissue-specific CpGs that would distinguish tumor from non-tumor tissues, the correlation analysis was used for the selection of highly tissue-specific biomarkers using 24 tumor samples with both DNAm and matched gene expression profiles (Fig. 1). Based on correlation analysis, 134 CpGs associated with 54 genes were obtained by integrating gene expression, DNAm, enzyme gene networks and gene co-expression networks. For genes with multiple CpGs, the CpGs with the highest correlation between DNAm and gene expression were selected (Table S6). The optimal number of features was determined by the automated searching algorithm of LASSO. Classifiers with higher complexity would contain redundant features with no corresponding improvement in performance, thereby resulting in poor generalization ability. Eventually, the five ccRCC-specific CpGs (Table 2) were obtained using the LASSO algorithm, and they were significantly differentially methylated between the tumor and matched adjacent normal tissues (*p*-value < 0.001; Wilcoxon rank sum test) (Fig. 4A). Unsupervised hierarchical clustering was further performed in the independent datasets, and the similarity matrix was constructed using Pearson’s correlation coefficient (Fig. 4B). Tumor tissues were well discriminated from the matched adjacent normal tissues in independent datasets, and an accuracy of 100% was obtained. The results showed that five CpGs had better robustness and the potential to distinguish cancer.

**Table 2:** Characteristics of five DNA methylation-based diagnostic biomarkers in ccRCC diagnosis.

Gene symbol	CpGs	Differential expression^a^ (log2FC)	Differential methylation^b^ level	Correlation^c^	Distance from TSS (bp)	CpG island
NAT8	cg19565262	2.73	−0.49	−0.46	−445	.
ALOX5	cg07355189	1.64	0.56	0.74	3,841	Island
PDE1A	cg00470341	−3.43	−0.53	0.74	53,972	.
PDE8B	cg12559197	−1.10	−0.55	0.70	1,48,031	.
PFKP	cg15087907	1.62	0.47	0.51	13,608	.

**Notes:**

a The log2-transformed fold changes between the tumor and benign-adjacent tissues (>0 means over-expression in tumor tissues) in ccRCCs.

b The differential methylation levels between the tumor and benign-adjacent tissues (>0 means hypermethylation in tumor tissues) in ccRCCs.

c The Spearman’s rank correlations between gene expression and probe methylation levels in ccRCCs.

### Functional enrichment and biological network analysis

**Figure 5:**
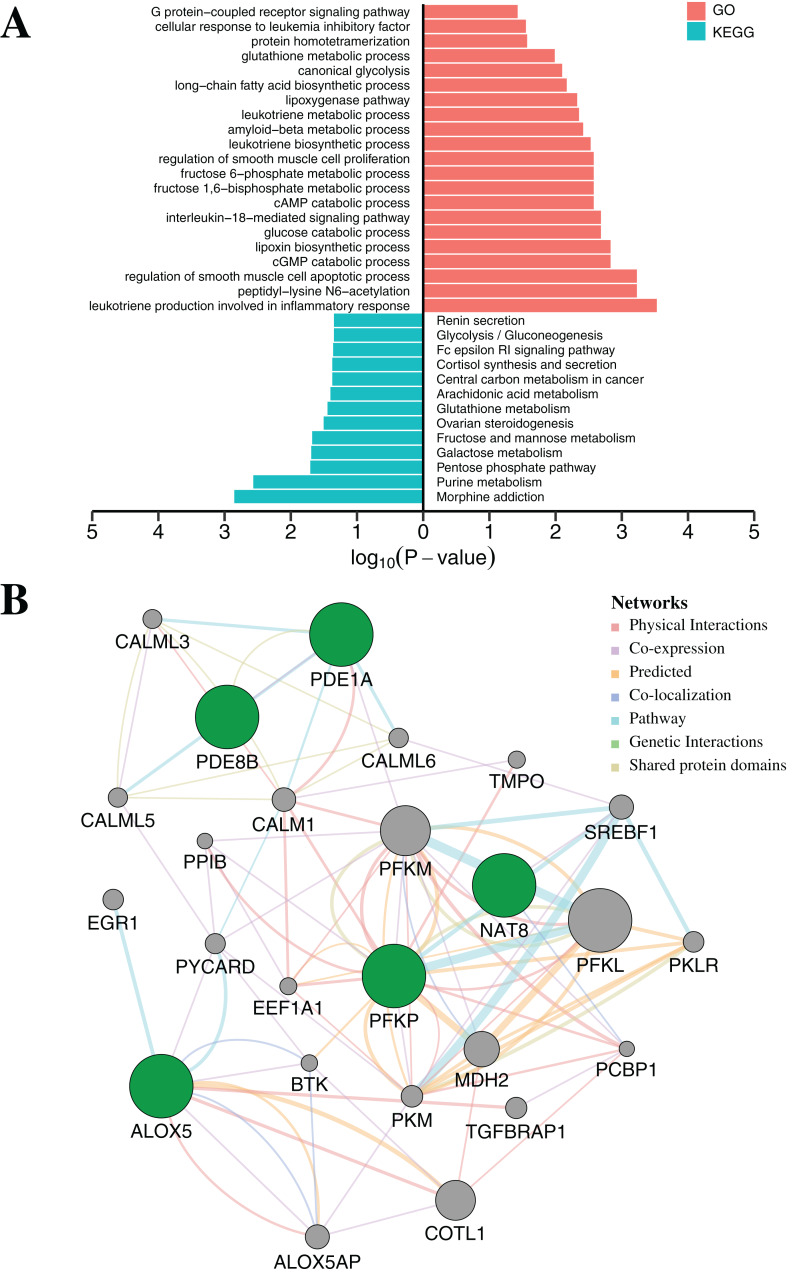
Functional enrichment analysis and protein–protein interaction network by DNA methylation-based diagnostic biomarkers. (A) Functional enrichment analysis was performed through GO and KEGG. *p*-Value was adjusted by Benjamini–Hochberg method. (B) The networks were each assigned a weight by the GeneMANIA algorithm. The weight of each edge was multiplied by weight of the containing network. The size of the circle was defined as the score attribute, which indicated the relevance of each gene to the original list based on the selected networks. Higher scores suggested that genes that were more likely to be functionally related. The shaded circles represented the DNA methylation-based diagnostic biomarkers.

To further elucidate the function of the above five CpGs significantly associated with gene expression, the molecular biological function analysis were performed by DAVID (Da Huang, Sherman & Lempicki, 2009) and *GeneMANIA* 3.6 (Warde-Farley et al., 2010). As shown in Fig. 5A (Table S7), the genes associated with five CpGs were mainly enriched in Morphine addiction, Purine metabolism, Pentose phosphate pathway, Galactose metabolism and Fructose and mannose metabolism. In general, the reprograming of cellular metabolism is a major hallmark of cancer cells, which is a direct and indirect consequence of carcinogenic factors. The common characteristic of cancer cell metabolism is the ability to access adequate nutrients from its surrounding environment and make good use of these nutrients to maintain viability or proliferation (Fouad & Aanei, 2017; Pavlova & Thompson, 2016). Two major nutrients utilized for survival and biosynthesis in cancer cells are glucose and glutamine. Interestingly, *PFKP* and *NAT8* were significantly associated with Glycolysis/Gluconeogenesis, Fructose and mannose metabolism, Galactose metabolism and Glutathione metabolism (*p*-value < 0.05, Fisher’s exact test). In addition, *PDE1A* and *PDE8B* were significantly enriched in Purine metabolism and G protein-coupled receptor signaling pathway (*p*-value < 0.05, Fisher’s exact test). Apart from playing an indispensable role in synthesizing DNA and RNA, purine metabolites supply some cancer cells with necessary nutrients and cofactors for survival or proliferation (Pedley & Benkovic, 2017; Yin et al., 2018) and they are also being targeted for the treatment of cancers (Fridley et al., 2011). Moreover, the majority of biological responses in both normal and cancer cells are regulated via multiple signaling pathways by G protein-coupled receptors (GPCRs) (Ristic, Bhutia & Ganapathy, 2017). Some research has suggested that GPCRs played an important role in purinosome assembly/disassembly and regulated metabolic flux through de novo purine biosynthesis in cells (Fang et al., 2013; Verrier et al., 2011). Furthermore, the *ALOX5* gene was associated with several physiological and pathological processes, including inflammation, oxidative response and carcinogenesis (Tomita et al., 2019). Protein–protein interaction networks from *GeneMANIA* 3.6 suggested a more complex interaction for *PFKP*, *PDE1A* and *ALOX5* (Fig. 5B). Most of these genes, including *SREBF1* (Audet-Walsh et al., 2018), *CALM1* (Fortney et al., 2015), *PCBP1* (Zhang et al., 2015), *COTL1* (Guo et al., 2017) and *BTK* (Yue et al., 2017), are tumor-related genes, which have been reported to be associated with tumorigenesis, progression and therapy.

### Evaluation and validation of diagnostic accuracy with 10-fold cross-validation

**Figure 6:**
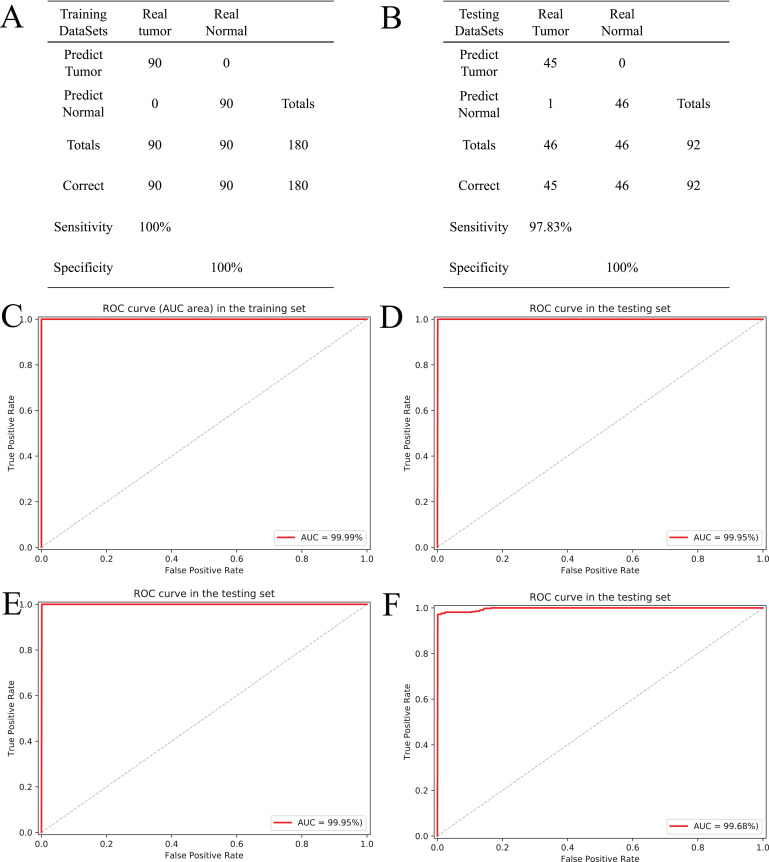
Construction and validation of the CpGs-based diagnostic model. (A and B) Confusion tables of binary results of diagnostic prediction model in the training (A) and validation (B). ROC of the diagnostic prediction model with methylation biomarkers in the training (C) and validation data sets (D). (E and F) ROC of the diagnostic prediction model with methylation biomarkers in two independent data sets (GSE70303 (E) and E-MTAB-2007 (F)).

Based on the five CpGs, a RF algorithm was implemented to construct an effectively diagnostic classifier. We obtained a sensitivity of 100% and specificity of 100% for ccRCC in the training dataset of 90 ccRCC and control samples (Fig. 6A) as well as a sensitivity of 97.83% and a specificity of 100% in the testing dataset of 46 ccRCC and control samples (Fig. 6B). Furthermore, the diagnostic classifier was demonstrated to effectively discriminate ccRCC from normal samples both in the training dataset (AUC = 99.99%) and the testing dataset (AUC = 99.95%) (Figs. 6C and 6D). To evaluate the performance of the five-CpG-based classifier, comparison of different independent datasets was required. In our research, two independent datasets, namely GSE70303 (Becket et al., 2016) and E-MTAB-2007 (Sato et al., 2013), were used with AUCs for 100% and 99.68% (Figs. 6E and 6F), respectively. To explore and verify universal applicability of the feature selection method and classifiers, the five most common cancers from TCGA were used to construct diagnostic classifiers, and the independent datasets from GEO were used to evaluate performance (GSE69914, GSE48684, GSE66836, GSE76938 and GSE54503). Remarkably, the AUCs based on the selected cancers were 97.31% (BRCA), 92.68% (COAD), 98.03% (LUAD), 94.90% (PRAD) and 98.32% (LIHC) (Fig. S5). The results showed that screening and identification of biomarkers based on multi-omics analysis could be extended to the application for any other cancers.

**Figure 7:**
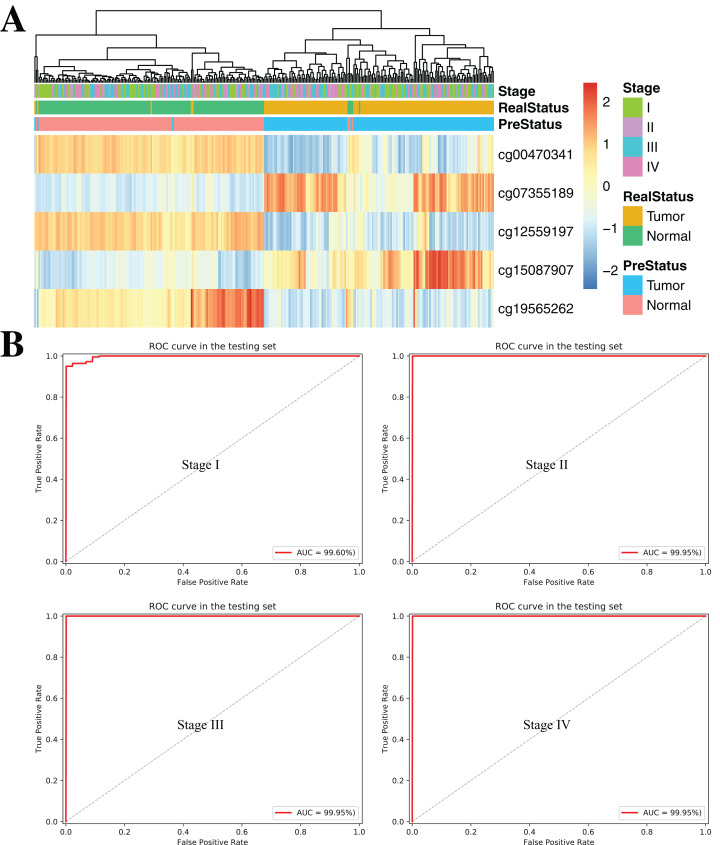
DNA methylation analysis of ccRCC diagnosis at different stages of tumorigenesis. (A) Unsupervised hierarchical clustering and heatmap for the methylation profile of the selected five CpGs across 160 samples at different stages of tumorigenesis. (B) ROC curve for the validation data sets of stages I–IV from TCGA.

To further demonstrate the diagnostic capacity of the five-CpG-based classifier in the early stages, the diagnostic classifier was implemented and evaluated at different cancer stages (stage I–IV). Firstly, the unsupervised hierarchical clustering was implemented in the independent datasets, including four different cancer stages. Strikingly, these patients were divided into two different groups, which separately contained tumor and non-tumor tissues (Fig. 7A). Subsequently, the five-CpG-based classifier was used to distinguish ccRCC from normal tissues at different stages. Remarkably, the five-CpG-based classifier achieved ROC curves with an AUCs of 99.60% (stage I) and 99.95% (stage II–IV) (Fig. 7B). The results indicated that the DNAm levels of the five CpGs were potentially effective biomarkers for distinguishing tumor tissues from normal tissues in the early stages (stage I–II).

### Constructing and validating the prognostic model

**Figure 8:**
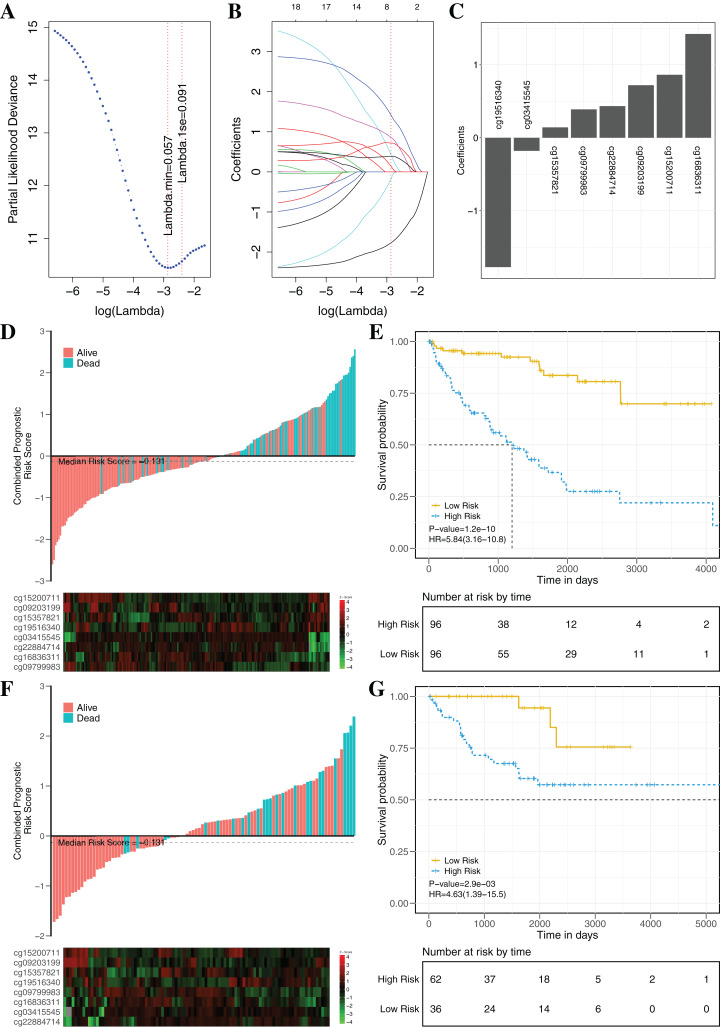
Construction and validation of the eight-CpG-based classifier. (A and C) Eight CpG sites selected by LASSO Cox regression analysis. (A) The two dotted vertical lines are drawn at the optimal values by minimum criteria and 1-s.e. criteria. Details are provided in Methods. (B) LASSO coefficient profiles of the 21 CpG sites. A vertical line is drawn at the optimal value by 1-s.e. criteria and results in eight non-zero coefficients. (C) A histogram of the absolute values of the coefficients for eight CpG sites, and eight CpG sites was selected in the LASSO Cox regression model. (D–G) Risk score was calculated by the eight-CpG-based classifier and Kaplan–Meier survival in the training data sets (D and E) and validation data set (F and G). Risk-score distribution of the eight-CpG-based classifier and patient survival status. Heatmap showing methylation of the eight CpG sites in the patients. Kaplan–Merier survival analysis for the patients. The patients were divided into low-risk and high-risk groups using the median cutoff value of the classifier risk score (−0.131). *p*-Values were calculated using the log-rank test. HR, hazard ratio.

To establish an effectively prognostic classifier for ccRCC, a series of rigorous criteria were used to filtrate biomarkers (see “Materials and Methods”). Table S8 showed that 21 CpGs were significantly correlated with overall survival based on univariate Cox regression analysis (*p*-value = 0.035 – 3.8 × 10^−6^). Subsequently, the multivariate LASSO Cox regression analysis was performed to screen prognostic biomarkers, and 8 of 21 CpGs were obtained (Figs. 8A–8C). Based on the β*-*values of the eight CpGs, the weighting coefficients were used to calculate risk scores for patients as follow: risk-score = −1.77 × cg19516340 + 0.43 × cg22884714 + 0.72 × cg09203199 + 1.42 × cg16836311 + 0.14 × cg15357821 −0.18 × cg03415545 + 0.86 × cg15200711 + 0.39 × cg09799983. The risk scores was used to evaluate survival status, patients with higher risk scores had poor survival status comparing with those with lower risk scores in two datasets (Figs. 8D and 8F). The median risk score (−0.13) was used as the cut-off, and patients were significantly grouped into high-risk and low-risk groups in the training datasets. The results showed that the risk-score was significantly correlated with risk of death (HR: 5.84; 95% confidence interval (CI) [3.16–10.8]; log-rank test *p*-value < 0.001; Fig. 8E). To assess the validity and reproducibility of prognostic classifier, patients in the validation datasets were also divided into high-risk or low-risk groups (HR: 4.63; CI [1.39–15.5]; log-rank test *p*-value = 2.9 × 10^−3^; Fig. 8G). The genes associated with the eight CpGs were identified, including *ADH1C*, *CES2*, *CYP1B1*, *LPCAT1*, *HOOK3*, *RRM2*, *CHEK2* and *MAN1C1*. The prognostic value of *RRM2*, *CES2*, *HOOK3*, *ADH1C* and *LPCAT1* has been reported and validated in many cancers, such as breast cancer, pancreatic cancer, colorectal cancer, bladder cancer, ovarian cancer, non-small cell lung cancer, head and neck cancer (Liu et al., 2007; Morikawa et al., 2010; Rahman et al., 2013; Xia et al., 2017; Yoshida et al., 2011). *MAN1C1* and *CHEK2* have been implicated in tumor immune response, cancer cell proliferation and cell-to-cell adhesion (Zhang et al., 2010), and provided potential therapeutic targets for RCC (Li et al., 2018). However, the molecular biological function of *CYP1B1* was still unknown. The results showed that those genes with abnormal methylation may paly crucial roles in tumorgenesis, and the DNAm sites might also be potential targets for new therapies.

The clinical prognostic signatures, including age, gender and TNM stage, were used as covariates to adjust prognostic classifier, and the results showed that the risk score calculated by the combination of eight CpGs was an independent prognostic factor with the better performance in the TCGA datasets (Table 3). Based on the subsets of ccRCC patients with different clinical signatures, the stratification analysis of the prognostic classifier was further implemented. When ccRCC patients were grouped according to the clinical signatures (age, gender, TNM stage), the prognostic classifier (Fig. S6) still had the better performance (log-rank test *p*-value < 0.05). The Fig. S7 shown that the ccRCC patients with the same clinical characteristics were also significantly divided into high-risk and low-risk groups by the prognostic classifier (log rank test *p*-value < 0.05). To further evaluate the universality of the construction methodology, we used five different cancers with the clinical information as the independent validation datasets (TCGA-BRCA, TCGA-KIRP, TCGA-LIHC, TCGA-LUAD and TCGA-UCEC). Compared to patients in the low-risk group, patients in the high-risk group had significantly shorter overall survival in all five different cancers (log-rank test, all *p*-value < 0.05; Fig. S8). In summary, these results clearly demonstrated that investigation of the DNAm mechanism with the multi-omics analysis may identify potential pathogenic factors and provide potential biomarkers for prognosis analysis of patients.

**Table 3:** Multivariate Cox regression analysis of the eight-CpG-based prognostic model with overall survival in the TCGA datasets.

Parameters		HR	(CI 95%) HR Lower	(CI 95%) HR Upper	SE	*Z* value	*p* Value
Gender	Male vs. Female	0.65	0.31	1.4	0.38	−1.10	*P* = 0.27
Age	Younger than 62 years versus 62 years or old	1.0	0.92	1.1	0.016	2.61	*P* = 0.011
pT	T1/2 versus T3/4	1.9	1.4	2.8	0.19	3.62	*P* < 0.001
pN	N0 versus N1	1.1	0.53	2.3	0.37	0.26	*P* = 0.8
M	M0 versus M1	1.4	0.78	2.5	0.31	1.10	*P* = 0.26
Stage	T1/2 versus T3/4	2.1	1.5	2.8	0.17	4.32	*P* < 0.001
Eight-CpG-Based model	Low vs. High risk groups	2.9	1.8	4.9	0.25	4.21	*P* < 0.001

**Note:**

HR, hazard ratio; CI, 95% confidence interval; SE, standard errors of coefficient; *Z* value, Wald *z*-statistic value.

## Discussion

Early diagnosis and intervention therapy of RCC were the most effective ways to reduce the death rate and prolong the life. As an epigenetic mechanism, the clinical value of DNAm changes was recognized widely (Linehan & Ricketts, 2019). In previous studies, researchers have mainly concentrated on the abnormal methylation of CGIs and promoter regions, but most of CpGs with abnormal methylation were located in the gene bodies, non-CGIs and intergenic regions (Jones, 2012). CpGs not in promoter regions may also have great effects on tumorigenesis and may offer patients help in terms of diagnosis or prognosis. The global human methylome screening performed in ccRCC patients (discovery cohort) analyzed using the Infinium HumanMethylation450 BeadChip array identified 4367 significantly hypermethylated sites (including 2,490 genes) and 5,031 significantly hypomethylated sites (including 3,551 genes) able to perfectly separate the ccRCC and non-ccRCC patients. These CpGs provided the basis for identifying diagnostic or prognostic biomarkers of ccRCC. In cancerous tissues, the hypermethylated CpGs were often located in the CGIs and closer to the TSS, while the hypomethylated CpGs were predominantly present in the non-CGIs. The molecular biological function analysis showed that these genes associated with abnormal methylation were associated with important biological processes, including cell adhesion, signal transduction, and transcriptional regulation. Findings also suggested that the abnormal DNAm was implicated in tumor initiation and progression

To further distinguish between potentially functional DNAm events (“driver events”) and cancer-free events (“passenger events”) (Selamat et al., 2012), DNAm and gene expression were integrated for comprehensively analysis. 68% of CpGs showed a negative correlation with gene expression. These CpGs were mainly found in promoter regions, which were associated with various cancerous mechanisms, such as cell migration and oncogenic transformation (Freitag et al., 2015). In the gene sets in which the methylation levels were positively correlated with gene expression profiles, approximately 10% of CpGs were also present in the promoter regions, suggesting that their mechanism of transcriptional regulation may be dominated by epigenetic modification other than DNAm. They were also closely related to tumorigenesis, such as *MUC15* (Huang et al., 2009), *HEPACAM2* (Klopfleisch et al., 2010), *CA10* (Romeo et al., 2009), *NRG1* (Huang et al., 2004) and *RAB25* (Mitra, Cheng & Mills, 2012), providing a novel information layer to our understanding of ccRCC. Another interesting finding was the dual activity of epigenetic mechanisms in ccRCC, such as *RUNX3* and *TMEM30B* (Fig. S4; Table S4). The existence of the “dual control” mechanism indicated that the change of DNAm levels located in the promoter may interact with those located in the gene body to regulate oncogene expression and tumorigenesis. Although the levels of DNAm and gene expression showed a “violation” of classical mechanisms of methylation-regulated expression, this phenomenon indicated that the regulation mechanisms of DNAm in ccRCC (even all cancers) were complexity. On the other hand, the abnormal methylation located in the gene bodies also played an important role in tumorigenesis, for example, *MUC15* (Huang et al., 2009), *HEPACAM2* (Klopfleisch et al., 2010), *CA10* (Romeo et al., 2009), *NRG1* (Huang et al., 2004) and *RAB25* (Mitra, Cheng & Mills, 2012), which was consistent with previous reports (Yang et al., 2014). The results indicated that gene body methylation not only offered novel insights for comprehensive understanding of DNAm mechanisms, but also provided new targets for cancer diagnosis and treatment. The Illumina HumanMethylation450 platform detected methylation sites based on probe hybridization and single nucleotide extension methods. This method of detecting DNAm levels using specific probes was highly dependent on the location of probe hybridization, such as CpGs located in the inner or outer of CGIs, and whether it was in close proximity to the TSS (Brenet et al., 2011; Van Vlodrop et al., 2011). In addition, the following properties also increased the complexity of regulation between DNAm and gene expression: methylation of distal regulatory elements; the distribution of DNAm sites (Clark, 2007); DNAm sites located at the edge of CGIs (“shores” and “shelves”) (Irizarry et al., 2009); DNAm in the regulation of alternatively spliced transcripts of the same gene (Maunakea et al., 2010); miRNA-based regulatory mechanism (Lopez-Serra & Esteller, 2012) and gene silencing due to the methylation of non-CGIs (Han et al., 2011). Therefore, the comprehensive detection of DNAm sites based on different techniques (such as whole-genome sulfite sequencing) will provide opportunities for the discovery of new methylation sites and regulatory mechanisms.

In addition to reinforcing or complementing the molecular mechanisms of gene expression, epigenetic integration also led to unexpected discoveries and therapeutic opportunities. During building the diagnostic or prognostic classifier, tumor biomarkers determined the performance in clinical practice. The redundant biomarkers tended to cause over-fitting of the classifier and resulted in poor generalization performance, while fewer biomarkers including incomplete or damaged information often resulted in under-fitting. Although DNAm was an important biological phenomenon in the development of many different types of cancer, there was a lack of greater insight into the physiological mechanisms of disease based solely on DNAm (Vrba et al., 2020; Zhou et al., 2018). Therefore, our multi-omics-based analysis provided a unified view for understanding the interrelationship between different molecular mechanisms and the combined effects on disease processes as well as for screening biomarkers with high sensitivity and specificity (Gao et al., 2019). At the end of this phase, 134 CpGs involving 54 genes were identified and might play an important role in carcinoma progression (Ristic, Bhutia & Ganapathy, 2017; Tomita et al., 2019). Subsequently, based on LASSO penalized regression or LASSO Cox regression, redundant features affecting model performance were further removed. Five CpGs for the diagnostic classifier and eight CpGs for the prognostic classifier were obtained, with the high sensitivity and specificity. More importantly, these specific biomarkers have been extensively studied in a variety of cancers, and involved in cell development, cell apoptosis, cancer metastasis or therapeutic response (Figs. 5A and 5B) (Clark, 2007; Lang et al., 2019; Van Vlodrop et al., 2011).

Unlike the small samples, the single-population samples or single-omics, this study utilized tumor samples from large databases (TCGA), which has the characteristic of owning large-size samples. Based on DNAm profiles, gene expression profiles, co-expression network and metabolic networks, the best features were selected to build the diagnostic or prognostic classifier. In clinical applications, the integration analysis of large samples, multi-population samples and multi-omics data greatly improved the reliability and universal application of diagnostic or prognostic classifiers. Based on the diagnostic classifier, an accuracy of 99.17% was obtained in the independent datasets. However, Wang et al. constructed a diagnostic classifier based on 44 gene expression profiles with an accuracy of 93.4% (Wang et al., 2017). Other researchers have established diagnostic classifiers based on mRNA or miRNA biomarkers with accuracies ranging from 90% to 96% (Spector et al., 2013; Youssef et al., 2011). However, there were some deficiencies in mRNA or miRNA, such as unstable expression, low sequence specificity and sensitivity, large amount and difficult to be found (Kwon et al., 2014). Our study also constructed a prognostic classifier, which effectively divided the patients into high-risk and low-risk groups. The prognostic classifier was a practical and powerful prognostic tool that provided prognostic value, complemented the current ccRCC staging system, and provided a theoretical basis for patient consultation and individual follow-up protocols.

## Conclusions

In summary, our study obtained novel biological insights when combining complementary epigenetic and transcriptomic analysis in the whole-genome perspective. Abnormal methylation events located in different genomic regions might have synergistic effects, which together caused abnormal expression patterns in metabolic pathways. The comprehensive analysis of genome-wide methylomic, transcriptomic and metabolic network may provide a promising avenue for facilitating the understanding of the mechanisms of ccRCC tumorigenesis. Based on the multi-level “omics” analysis, we also identified cancer-specific epigenetic signatures to diagnose and stratify patients. In addition, due to the reversibility of DNAm, they will also become the most promising therapeutic targets.

## Supplemental Information

10.7717/peerj.9654/supp-1 Supplemental Information 1 Characteristics of subjects and tumors.

10.7717/peerj.9654/supp-2 Supplemental Information 2 Gene exhibiting abnormal methylation and expression in ccRCC.

10.7717/peerj.9654/supp-3 Supplemental Information 3 Top 10 Gene Ontology (GO) biological process (BP) based on the genes with abnormal methylation.

10.7717/peerj.9654/supp-4 Supplemental Information 4 Genes exhibiting tandem promoter and gene body methylation from TCGA-KIRC datasets.

10.7717/peerj.9654/supp-5 Supplemental Information 5 Genes exhibiting tandem promoter and gene body methylation from GEO (KIRC) datasets.

10.7717/peerj.9654/supp-6 Supplemental Information 6 Distribution of differentially methylated CpG sites and genes in the feature selection stage.

10.7717/peerj.9654/supp-7 Supplemental Information 7 Function enrichment and biological network analysis using five CpG sites.

10.7717/peerj.9654/supp-8 Supplemental Information 8 Characters of 21 methylation markers and their coefficients in ccRCC.

10.7717/peerj.9654/supp-9 Supplemental Information 9 Unsupervised clustering of ccRCC and matched adjacent normal using DNA methylation patterns. The heat-map represents 160 ccRCC and 160 matched adjacent normal samples clustered using 28271 significantly differentially methylated CpG sites demonstrating the reliability of our method across the entire cohort of 320 primary samples. The majority of ccRCC (red, 99.38%) cluster together, as do the matched adjacent normal samples (blue, 100%).

10.7717/peerj.9654/supp-10 Supplemental Information 10 Identification of significantly differentially methylated CpG sites in the human genomes. G01, whether or not CpG sites were located in promoters; G02, whether or not CpG sites were located in CpG islands (CGIs). The promoter regions were defined as ±1.5kb from TSS, and the others were non-promoter regions.

10.7717/peerj.9654/supp-11 Supplemental Information 11 Intersection of genes with DNA methylation and gene expression profiles. A total of 6,041 (4,625 + 1,100 + 316) genes were profiled on the methylation array and a total of 2,478 (1,062 + 1,100 + 316) genes profiled on the expression array fulfilled the expression intensity filtering criteria, of which 1,416 genes overlap between the two platforms. The central set of 316 genes possessing a) Methylation and gene expression profiles, and b) exhibiting differential methylation between ccRCC tumors and matched adjacent normal tissues was used in the global methylation-expression correlation analysis. CpG sites targeting the X and Y chromosomes were removed prior to performing the differential methylation analysis, so all 316 genes applied to the global correlation analysis are located on autosomes.

10.7717/peerj.9654/supp-12 Supplemental Information 12 RUNX3 (left) and TMEM30B (right) are shown as examples. For each gene, data are drawn from the individual gene body and promoter CpG sites exhibiting the strongest absolute correlations with expression. (A) Relationship between expression intensity and gene body DNA methylation level. (B) Relationship between expression intensity and promoter DNA methylation level. (C) Relationship between gene body and promoter DNA methylation levels, confirming occurrence in the same samples. (D) Schematic representation of promoter and gene body methylation relationships to gene expression alterations in tumor samples relative to nonmalignant samples.

10.7717/peerj.9654/supp-13 Supplemental Information 13 Construction and validation of the probes-based diagnostic model. ROC curve for the validation datasets of GSE (BRCA), GSE (COAD), GSE (LIHC), GSE (LUAD) and GSE (PRAD).

10.7717/peerj.9654/supp-14 Supplemental Information 14 The analysis of Hazard ratio (HR) for TCGA datasets with ccRCC using the eight-CpG-based model in different subgroups stratified by clinical parameters.

10.7717/peerj.9654/supp-15 Supplemental Information 15 Kaplan–Meier survival analysis based on the eight-CpG-based model in subsets of different clinical stage patients with ccRCC (log-rank test).

10.7717/peerj.9654/supp-16 Supplemental Information 16 Construction of the probes-based prognostic classifier. Kaplan–Meier survival analysis of the patients in each of the five cancers. The patients were divided into low-risk and high-risk groups using the median cutoff value of the partial hazard. *p*-value were calculated by the log-rank test

## Additional Information and Declarations

### Microarray Data Deposition

The following information was supplied regarding the deposition of microarray data: Raw data are available at NCBI GEO: GSE70303, GSE69914, GSE48684, GSE66836, GSE76938 and GSE54503, and at ArrayExpress: E-MTAB-2007.

### Data Availability

The following information was supplied regarding data availability: The publicly available datasets analyzed in this study can be found in The Cancer Genome Atlas (https://portal.gdc.cancer.gov/) with the following search terms: TCGA-BRCA, TCGA-COAD, TCGA-LIHC, TCGA-LUAD, TCGA-PRAD, TCGA-KIRC, TCGA-KIRP and TCGA-UCEC.
