# Supplementary figures and images for: Identification of DNA methylation patterns and biomarkers for clear-cell renal cell carcinoma by multi-omics data analysis
Source: PeerJ. 2020 Aug 3;8:e9654. doi: 10.7717/peerj.9654 (PMC7409785; doi:10.7717/peerj.9654)

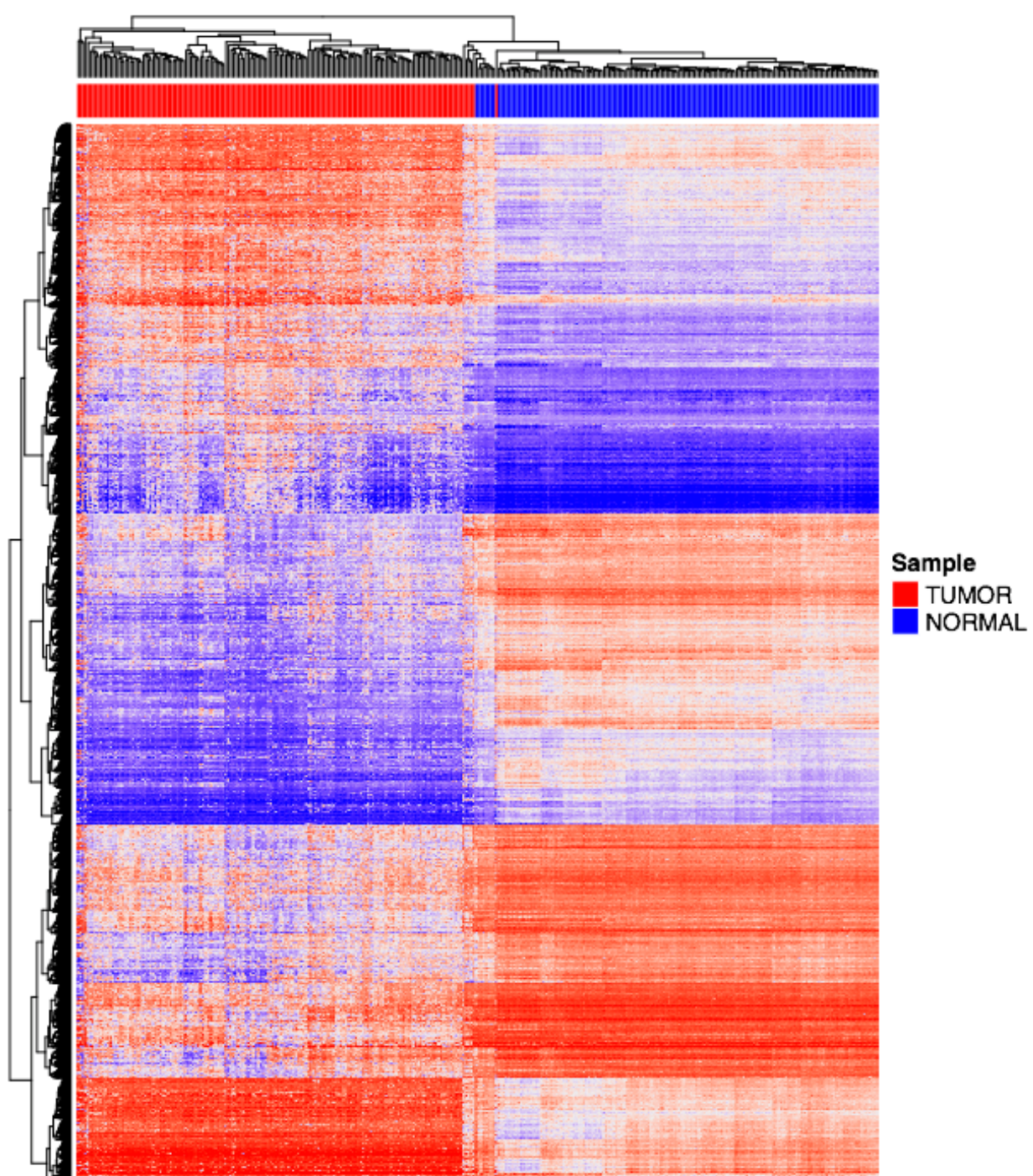

Supplement: Supplemental Information 9 — The heat-map represents 160 ccRCC and 160 matched adjacent normal samples clustered using 28271 significantly differentially methylated CpG sites demonstrating the reliability of our method across the entire cohort of 320 primary samples. The majority of ccRCC (red, 99.38%) cluster together, as do the matched adjacent normal samples (blue, 100%). [file peerj-08-9654-s009.pdf]

G01

Hypermethylation

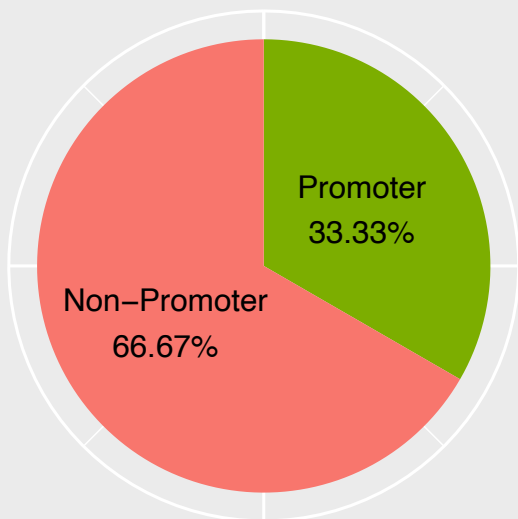

G01

Hypomethylation

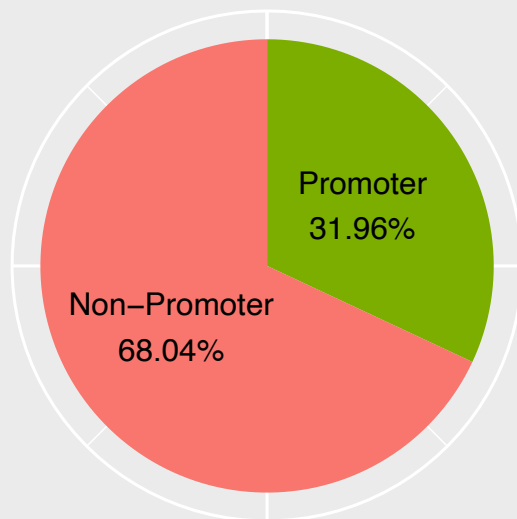

G02

Hypermethylation

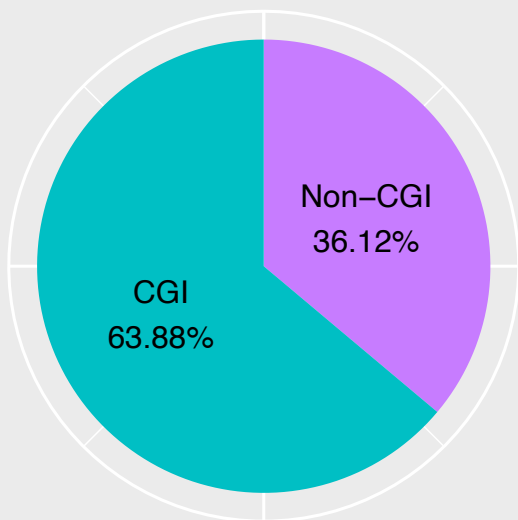

G02

Hypomethylation

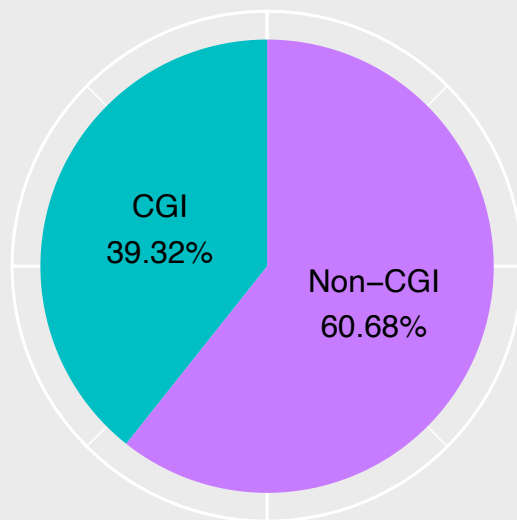

Supplement: Supplemental Information 10 — G01, whether or not CpG sites were located in promoters; G02, whether or not CpG sites were located in CpG islands (CGIs). The promoter regions were defined as ±1.5kb from TSS, and the others were non-promoter regions. [file peerj-08-9654-s010.pdf]

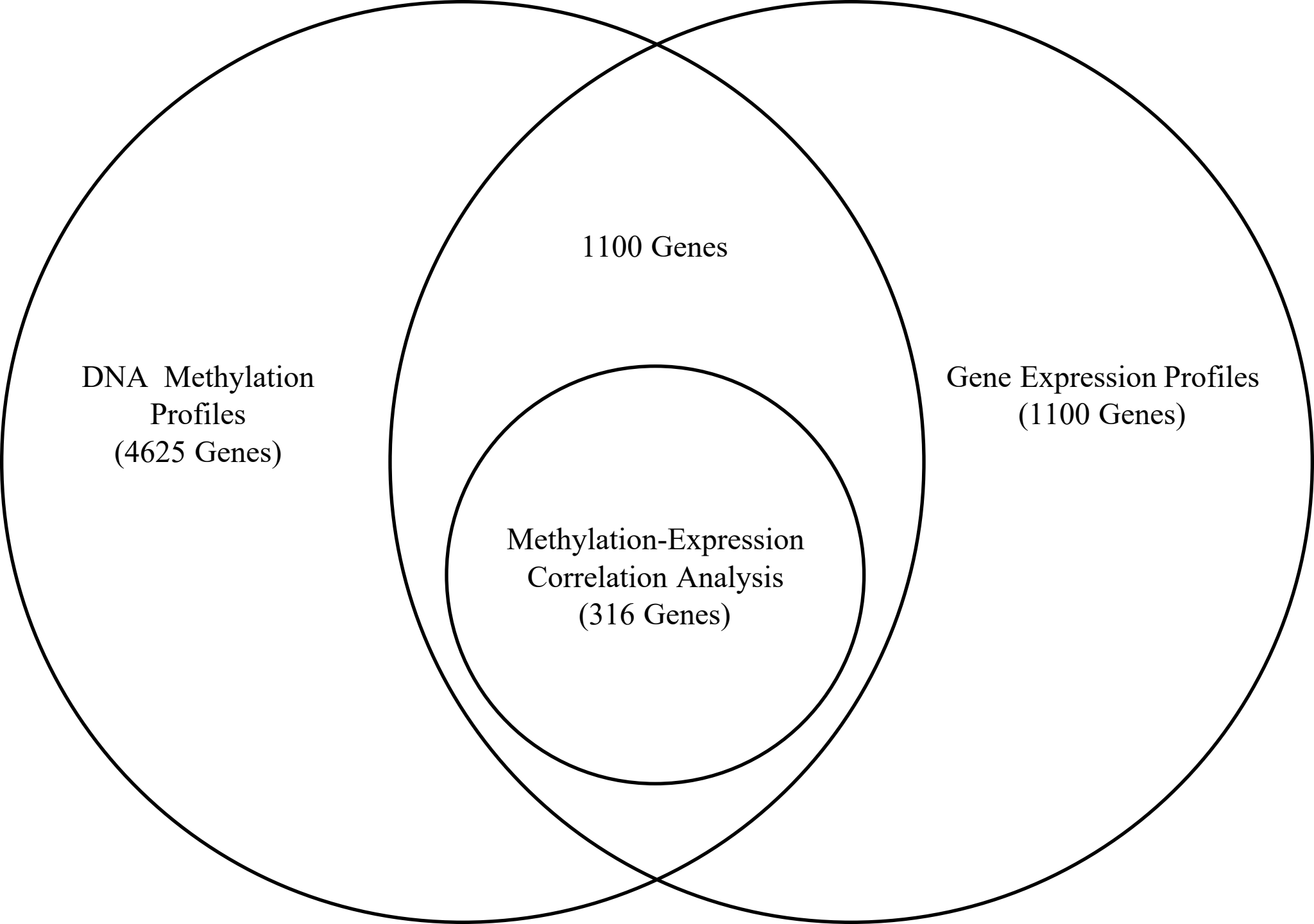

Supplement: Supplemental Information 11 — A total of 6,041 (4,625 + 1,100 + 316) genes were profiled on the methylation array and a total of 2,478 (1,062 + 1,100 + 316) genes profiled on the expression array fulfilled the expression intensity filtering criteria, of which 1,416 genes overlap between the two platforms. The central set of 316 genes possessing a) Methylation and gene expression profiles, and b) exhibiting differential methylation between ccRCC tumors and matched adjacent normal tissues was used in the global methylation-expression correlation analysis. CpG sites targeting the X and Y chromosomes were removed prior to performing the differential methylation analysis, so all 316 genes applied to the global correlation analysis are located on autosomes. [file peerj-08-9654-s011.pdf]

RUNX3

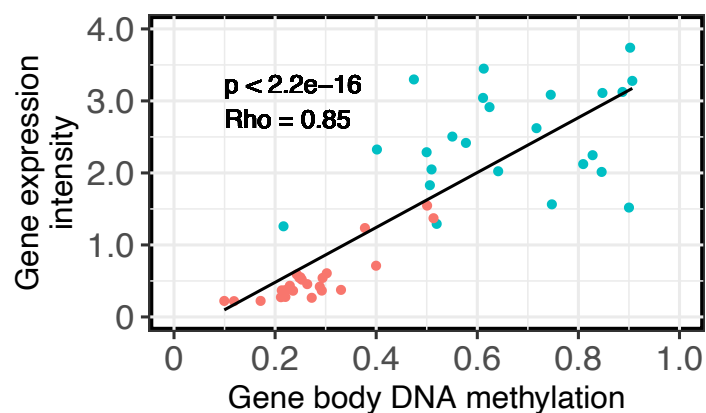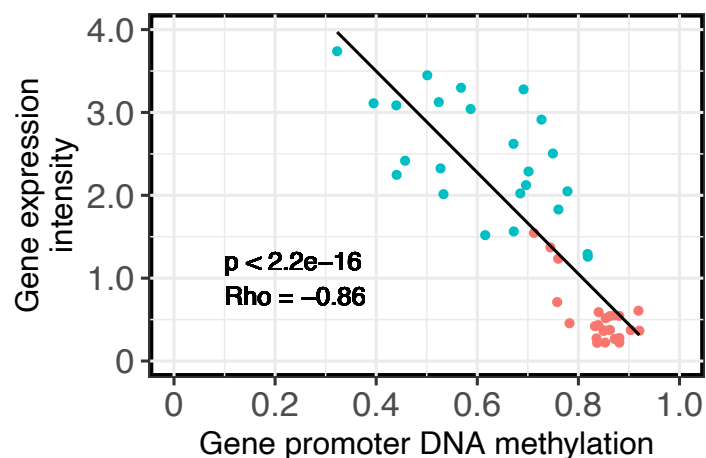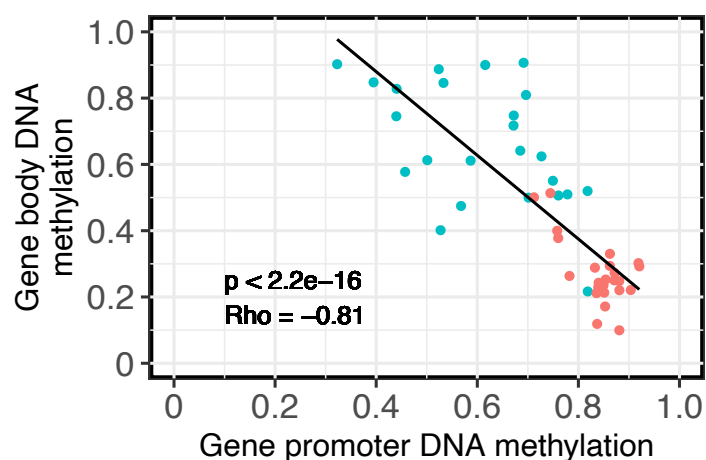

TMEM30B

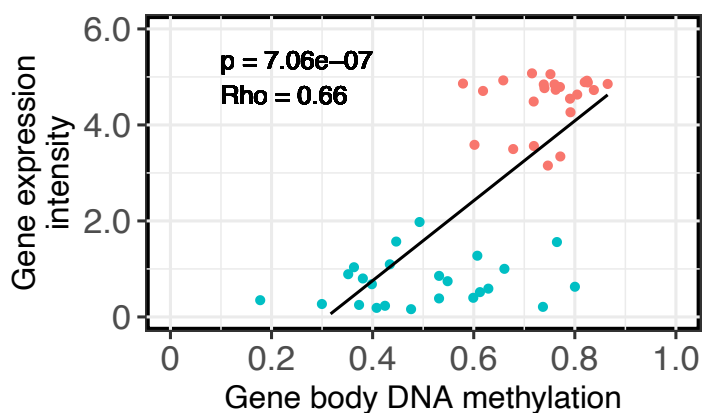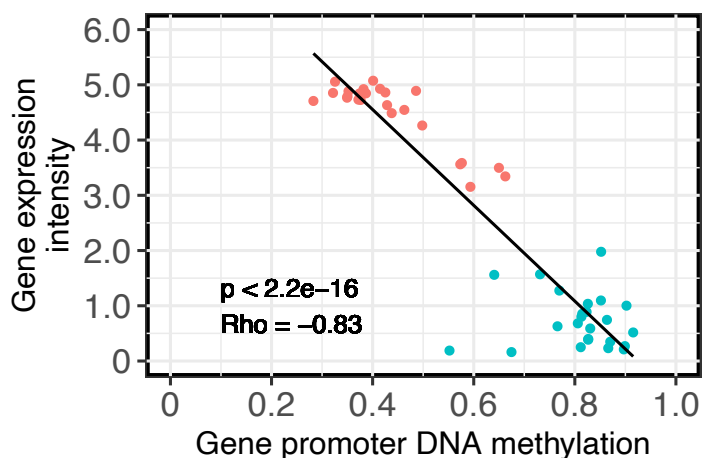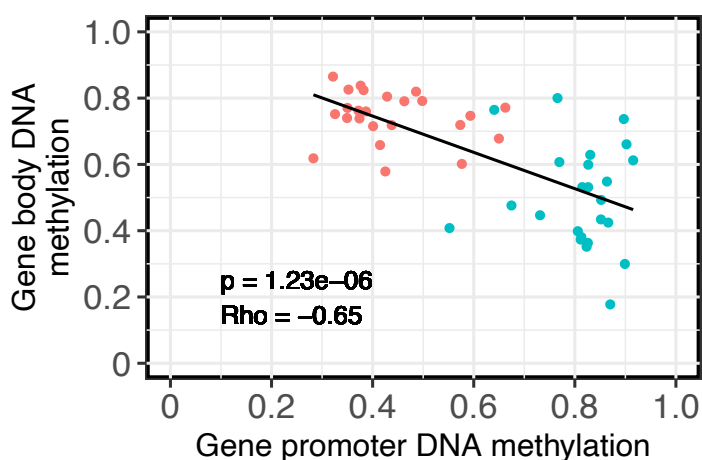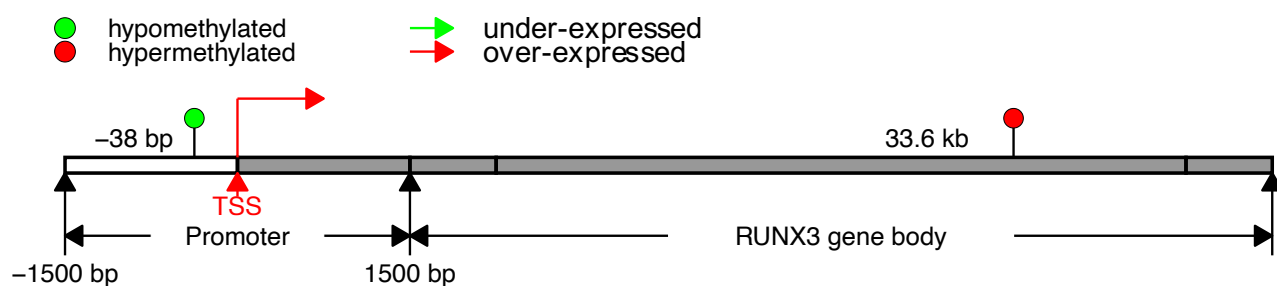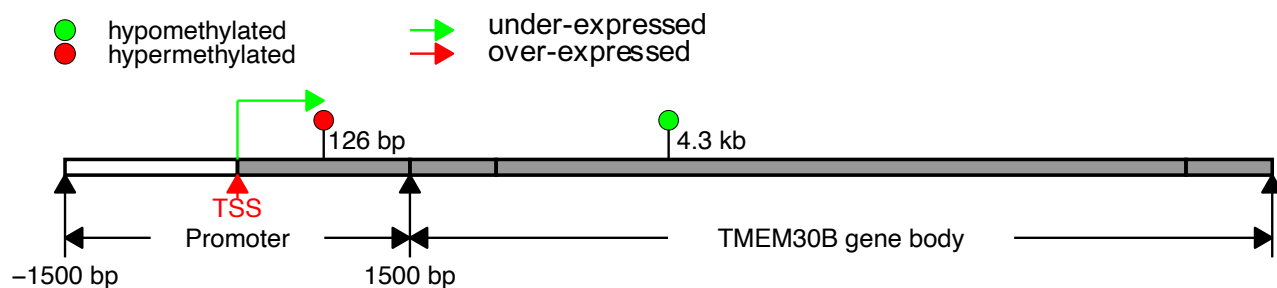

Supplement: Supplemental Information 12 — For each gene, data are drawn from the individual gene body and promoter CpG sites exhibiting the strongest absolute correlations with expression. (A) Relationship between expression intensity and gene body DNA methylation level. (B) Relationship between expression intensity and promoter DNA methylation level. (C) Relationship between gene body and promoter DNA methylation levels, confirming occurrence in the same samples. (D) Schematic representation of promoter and gene body methylation relationships to gene expression alterations in tumor samples relative to nonmalignant samples. [file peerj-08-9654-s012.pdf]

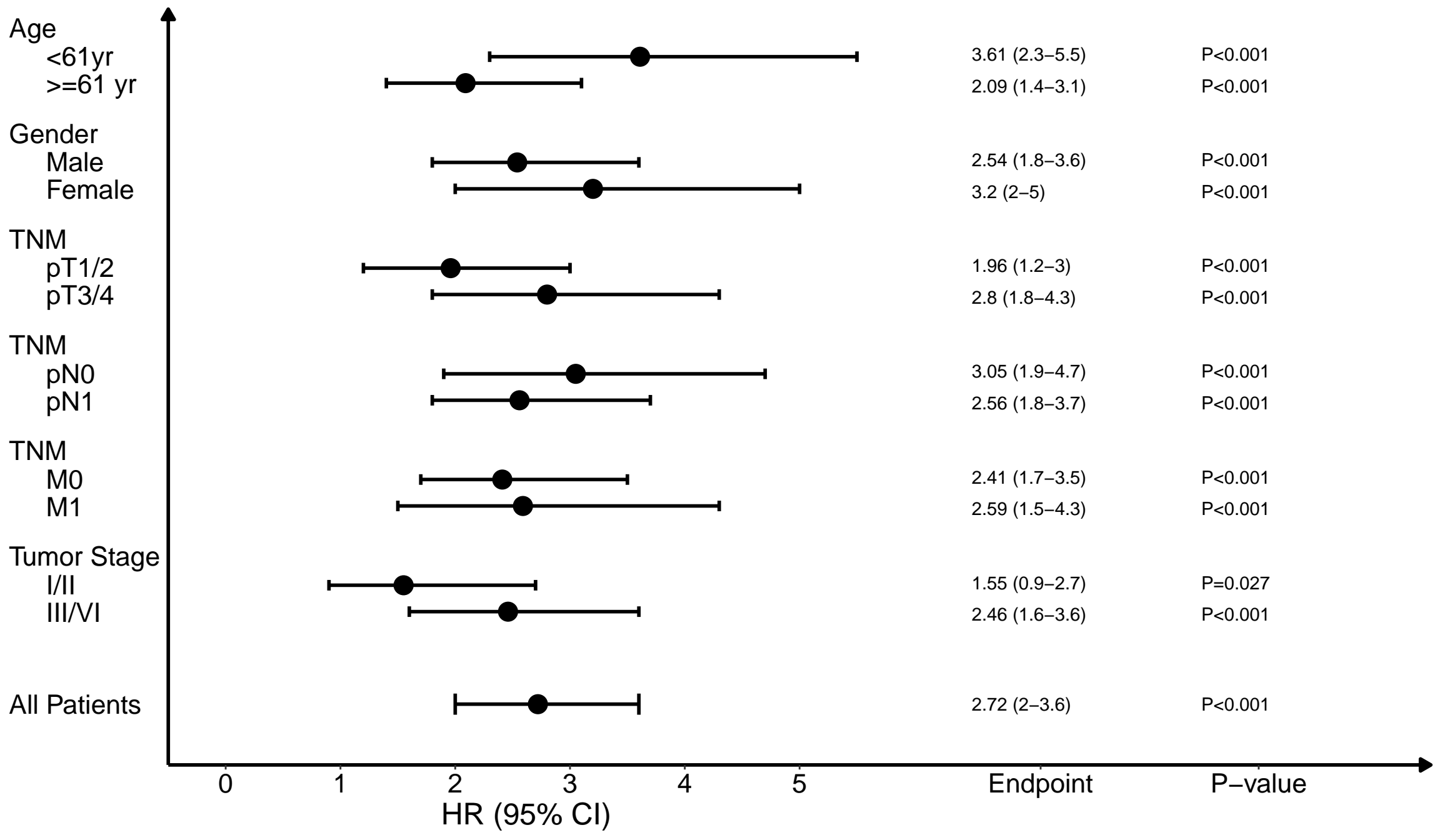

Supplement: Supplemental Information 14 [file peerj-08-9654-s014.pdf]

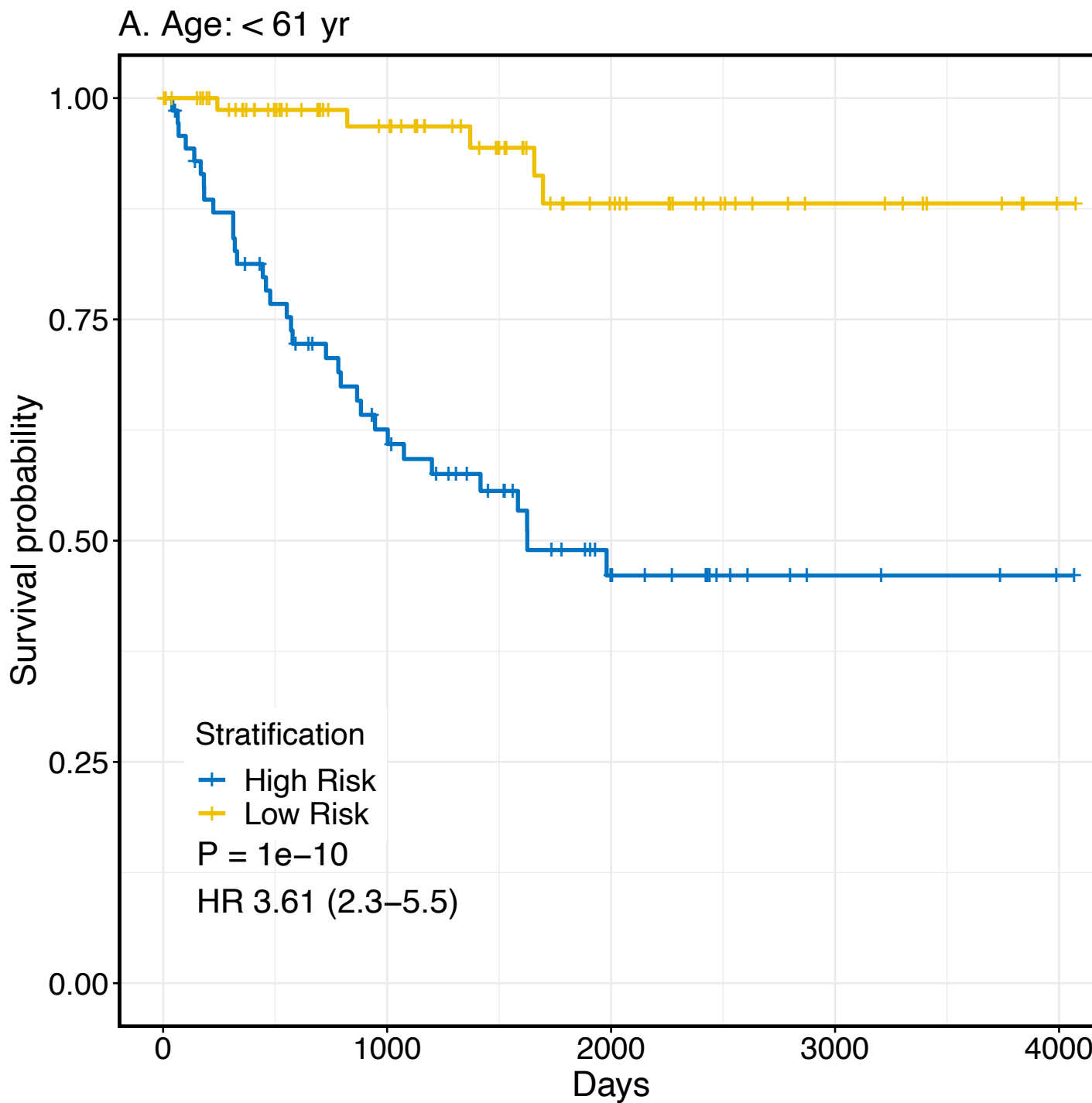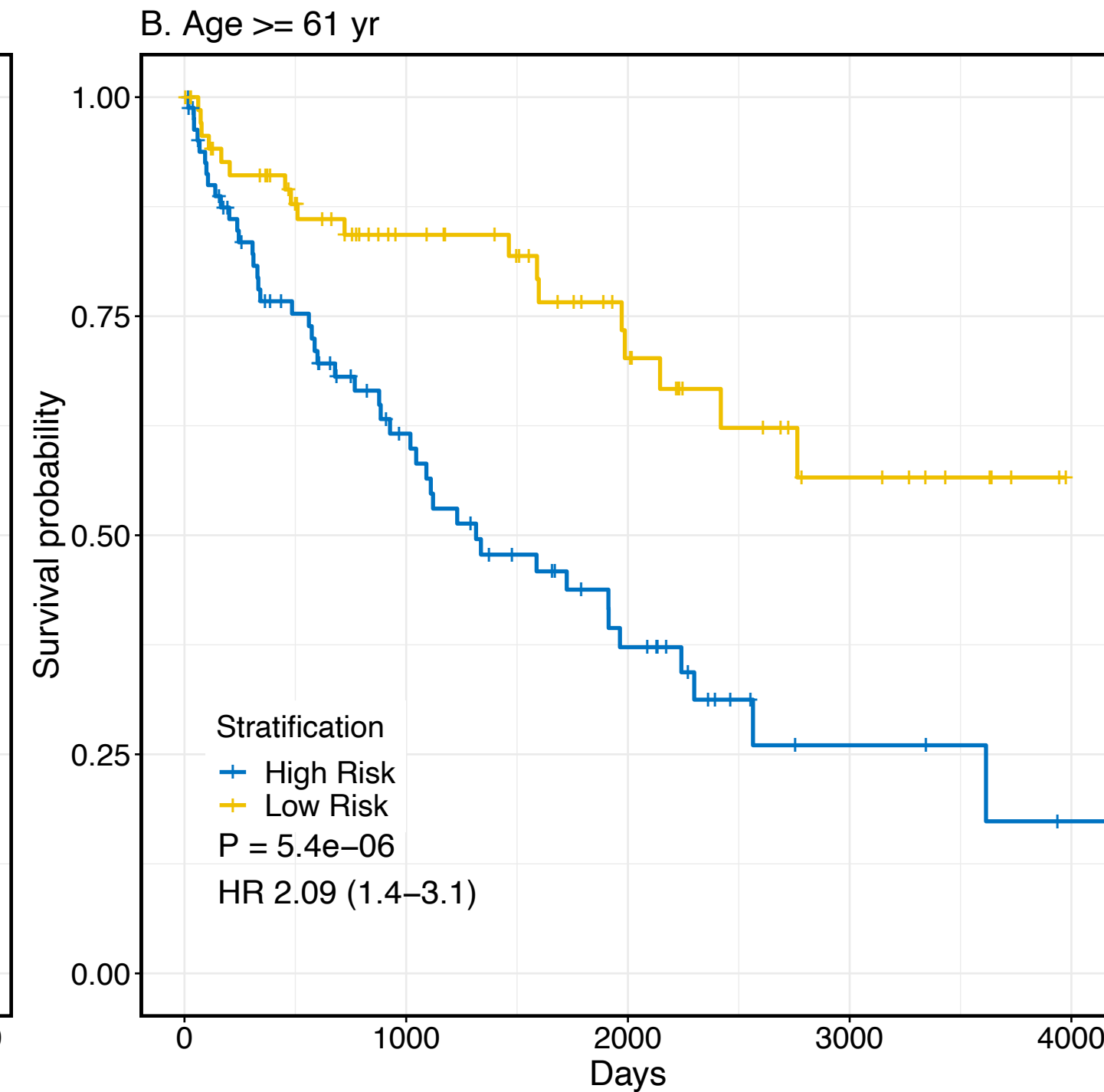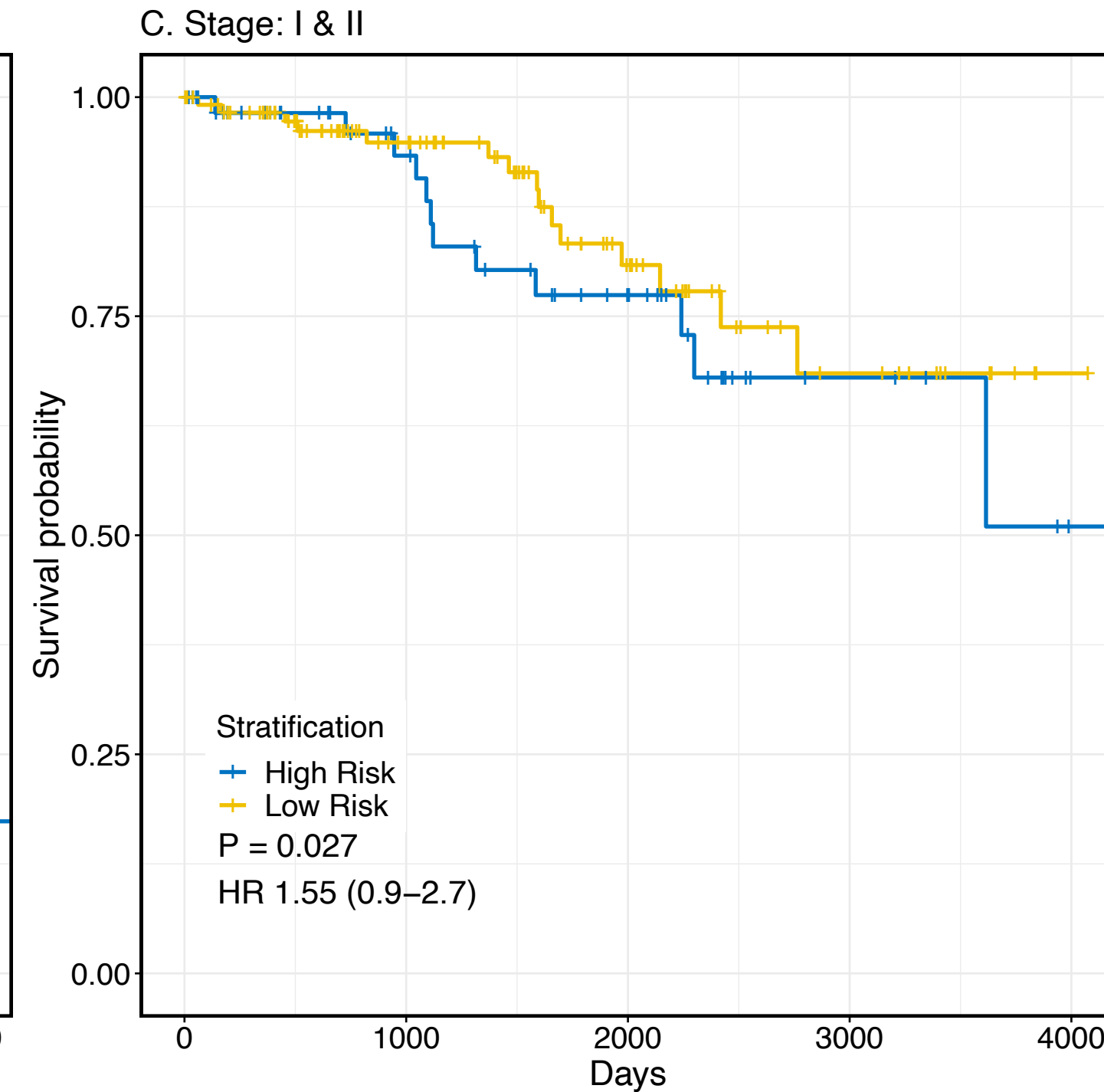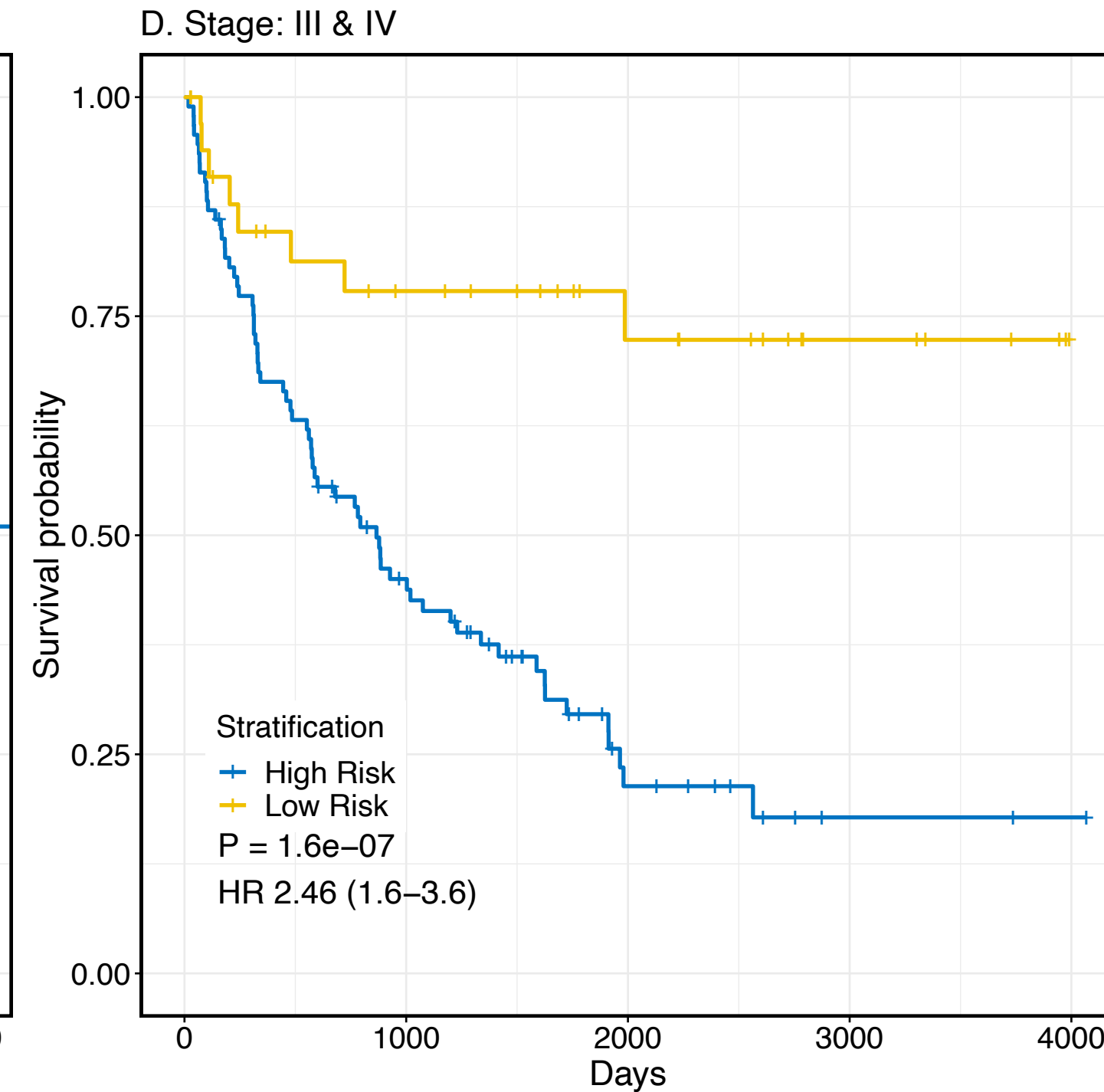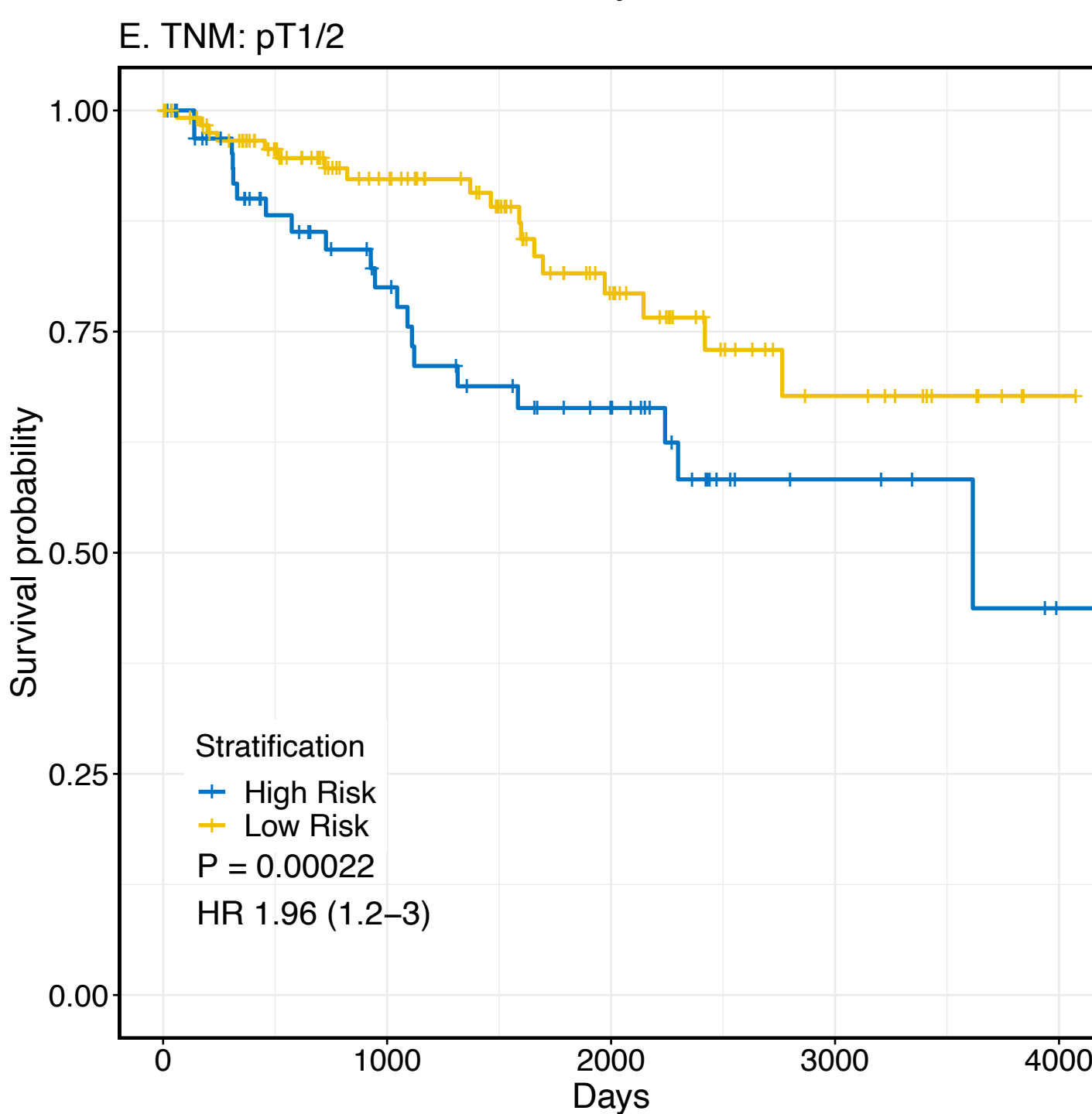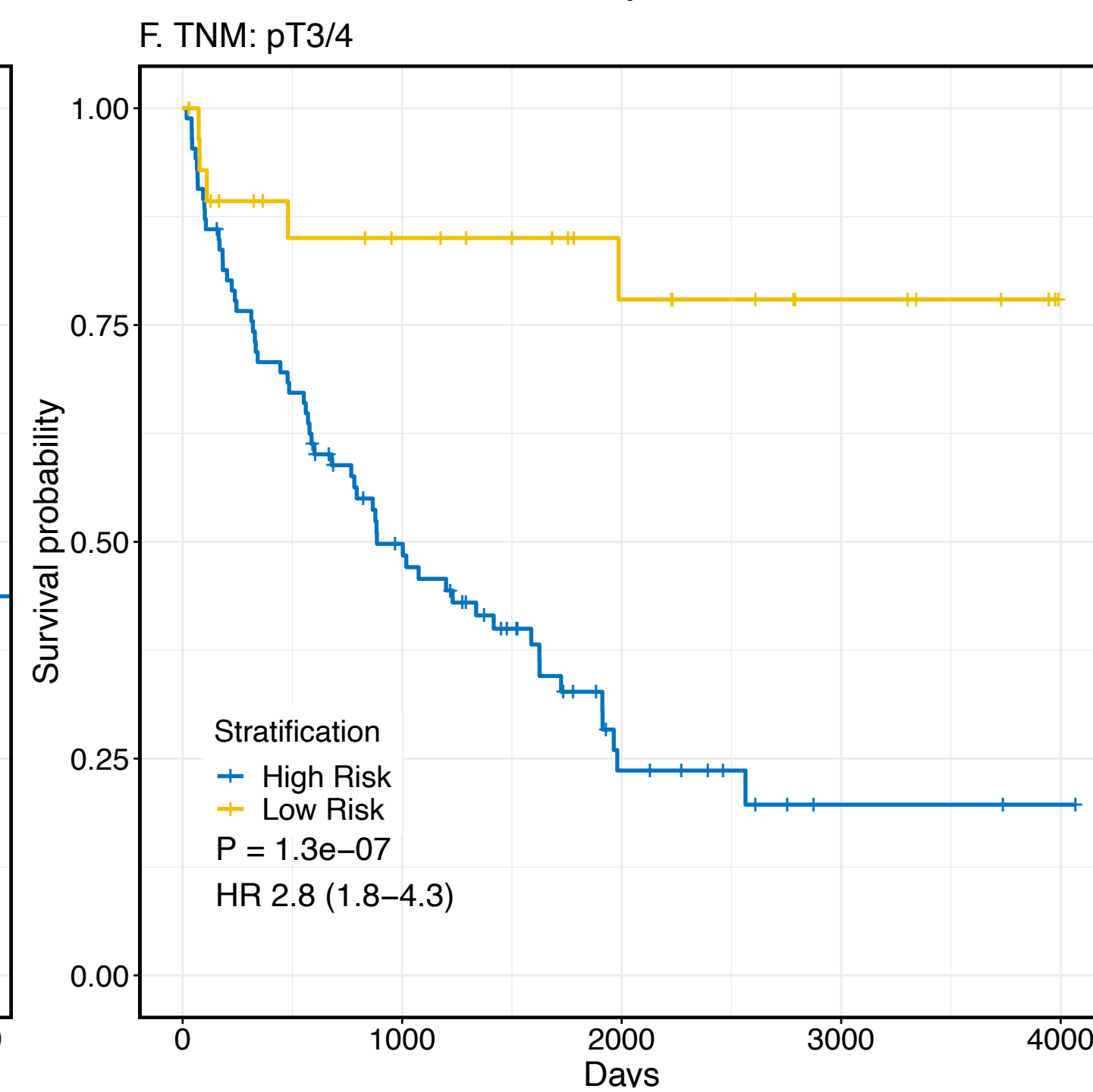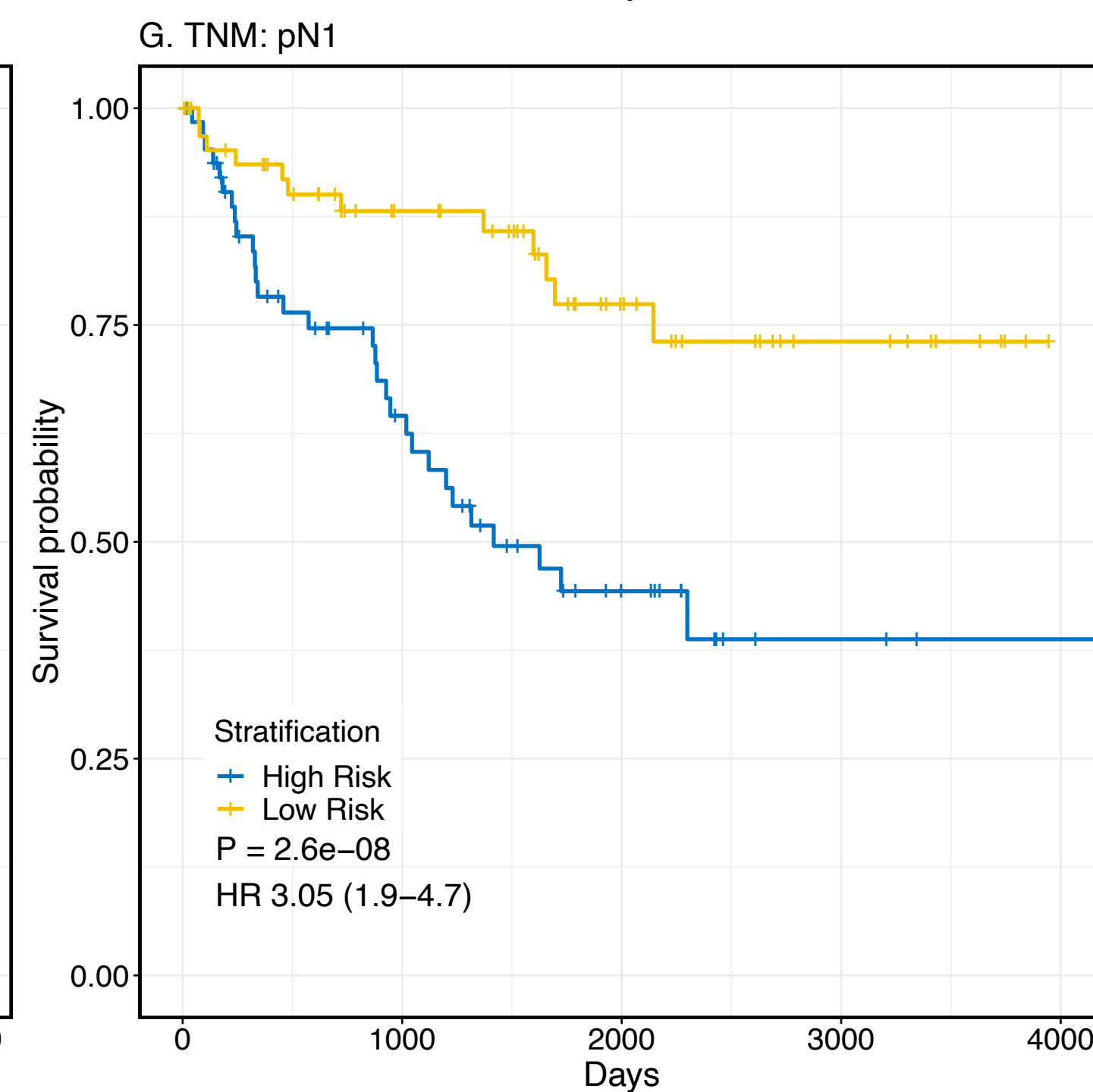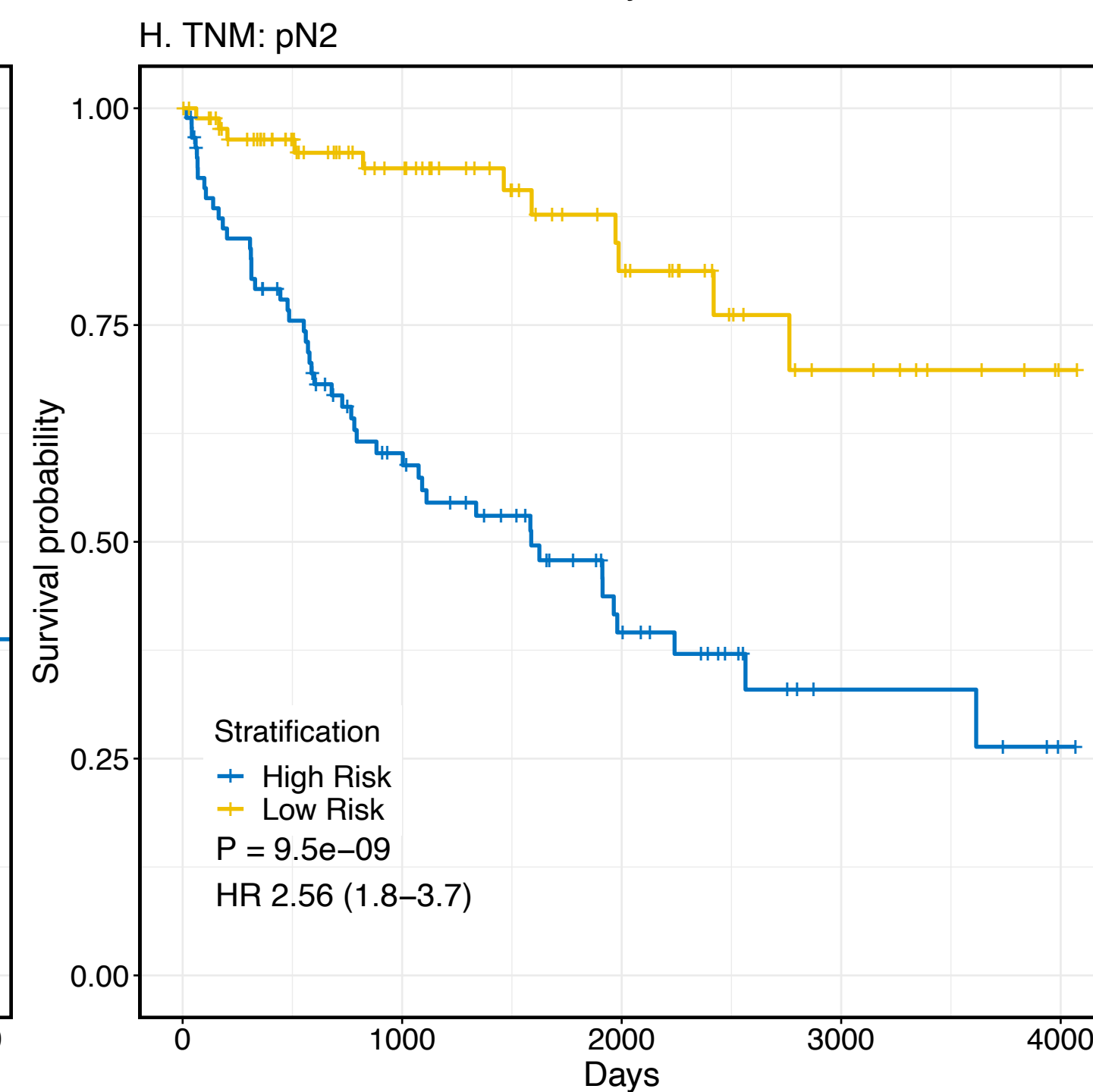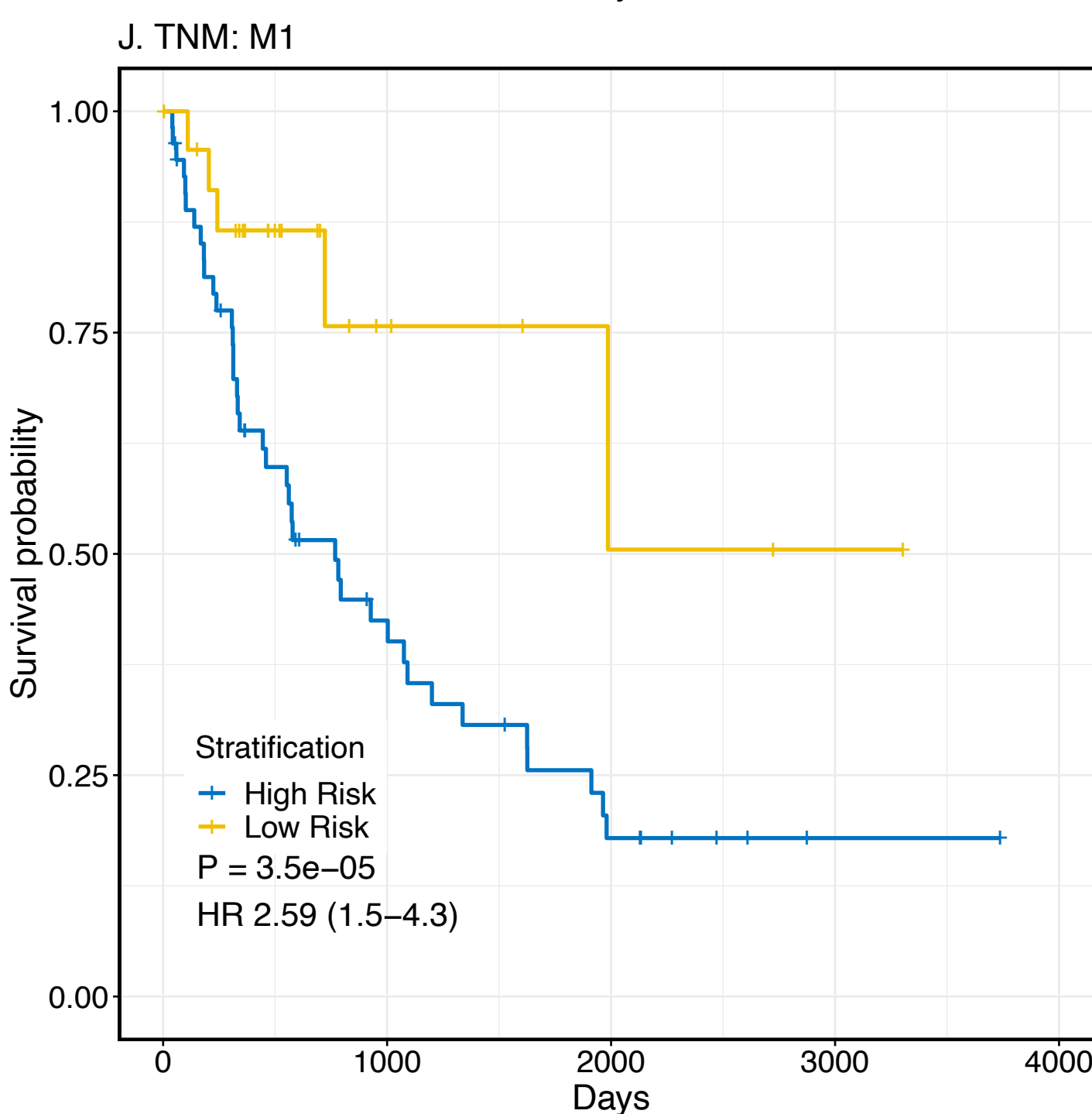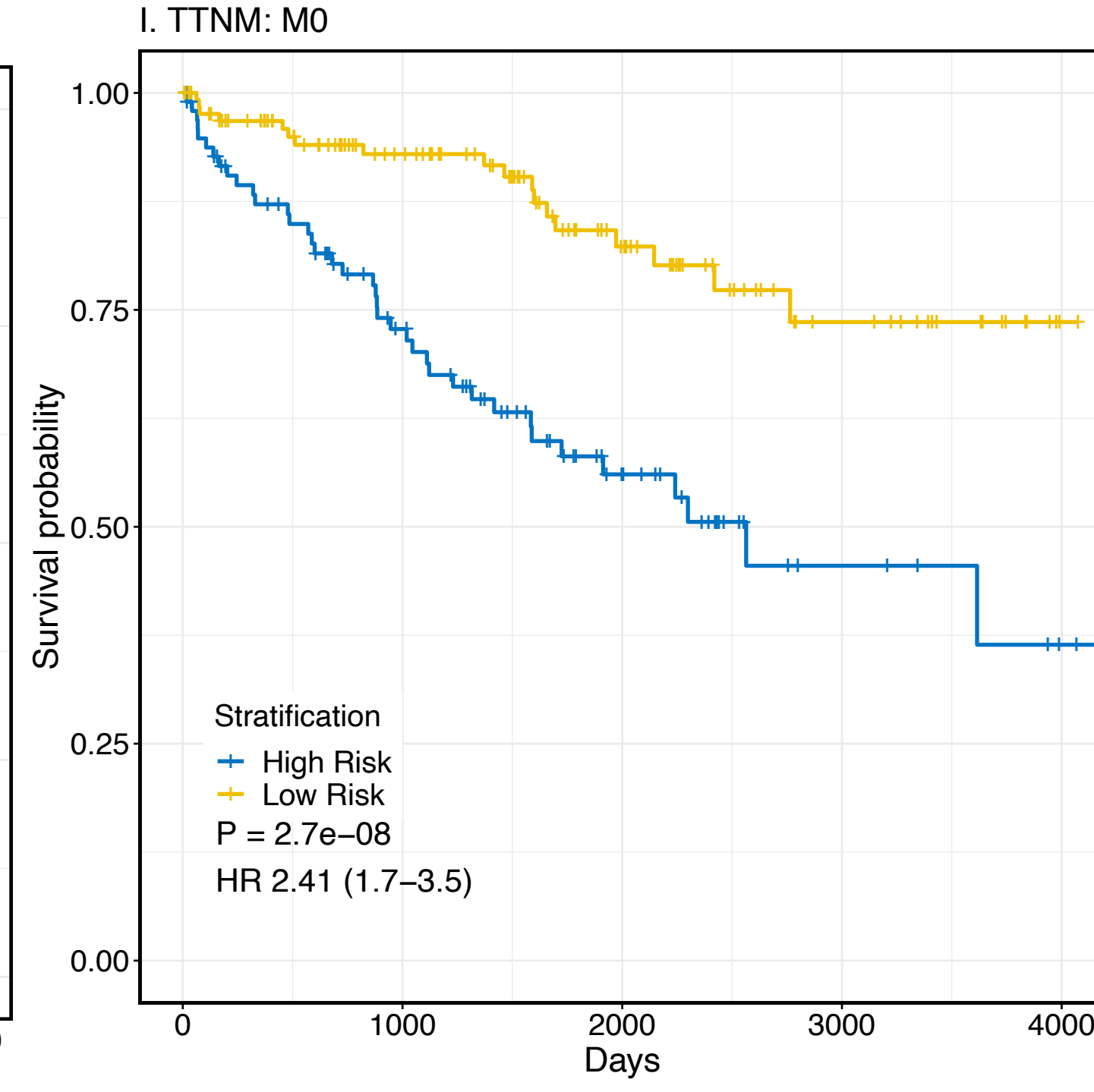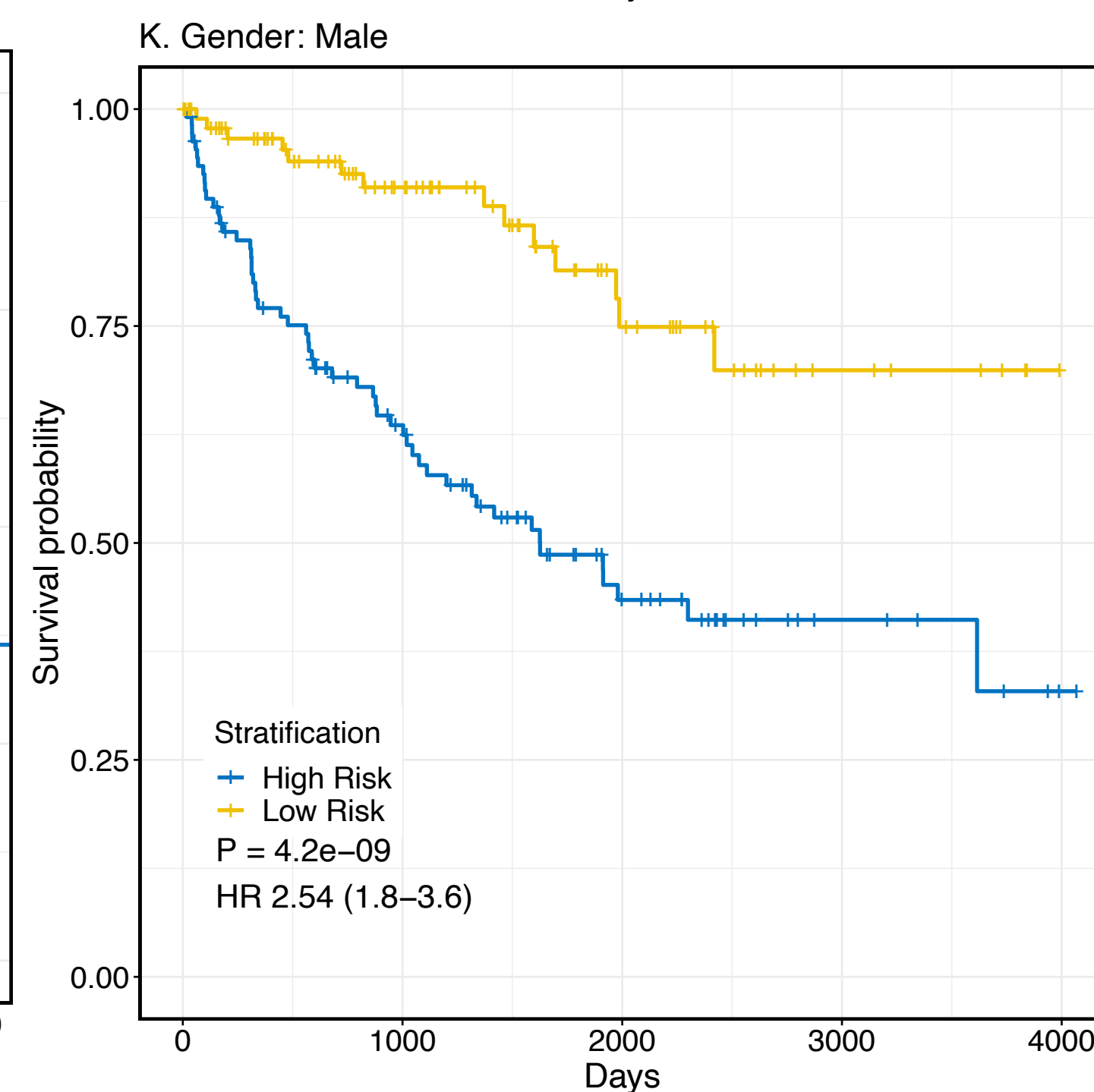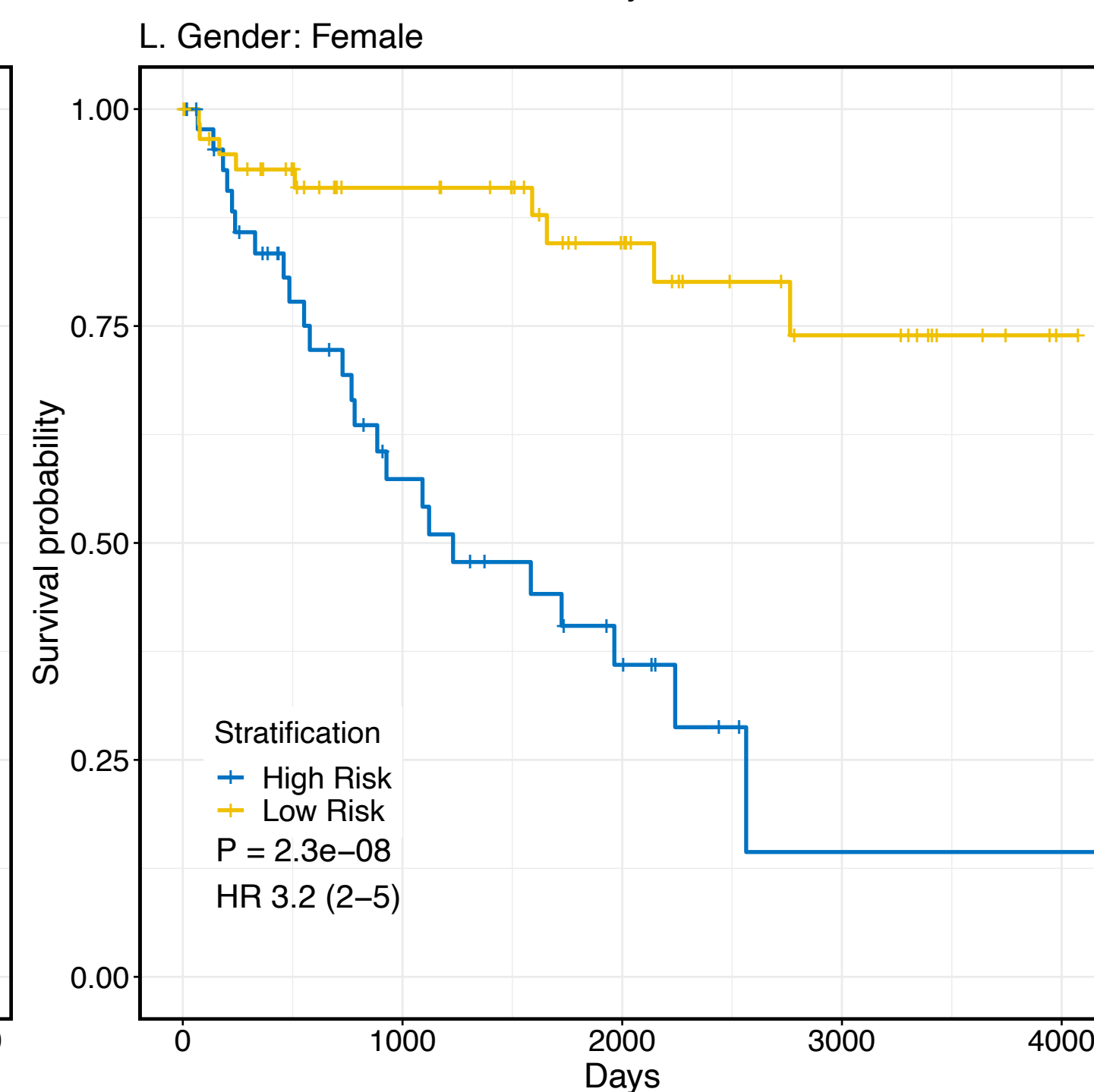

Supplement: Supplemental Information 15 [file peerj-08-9654-s015.pdf]

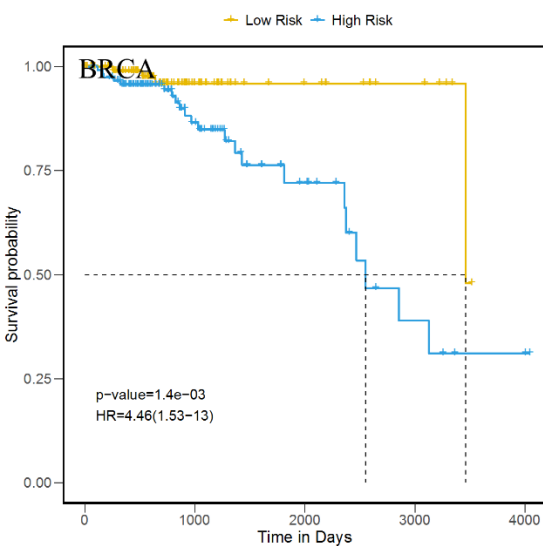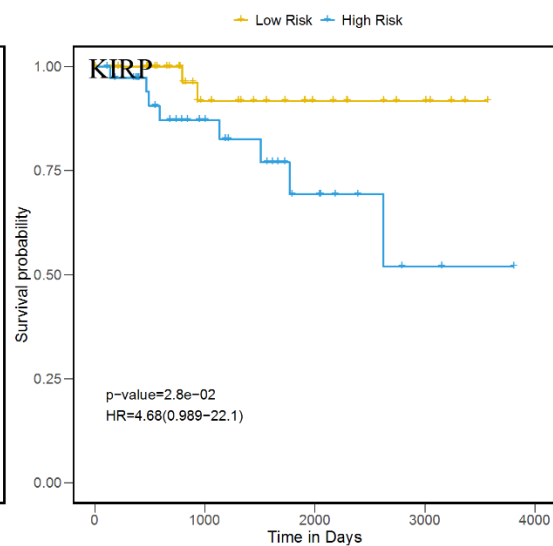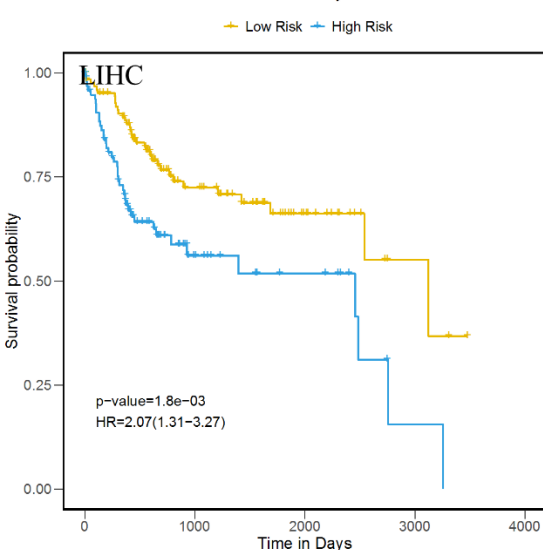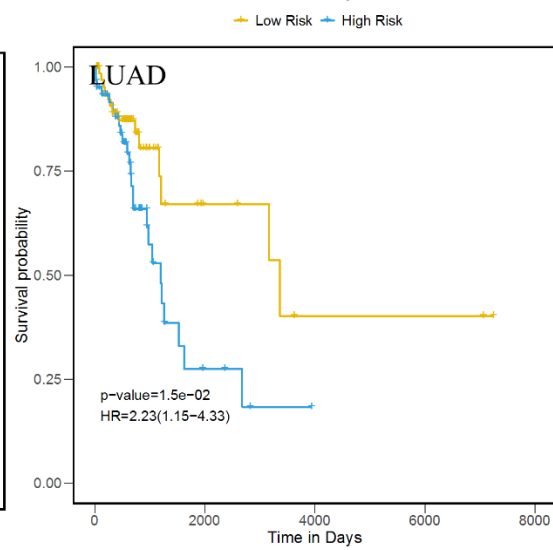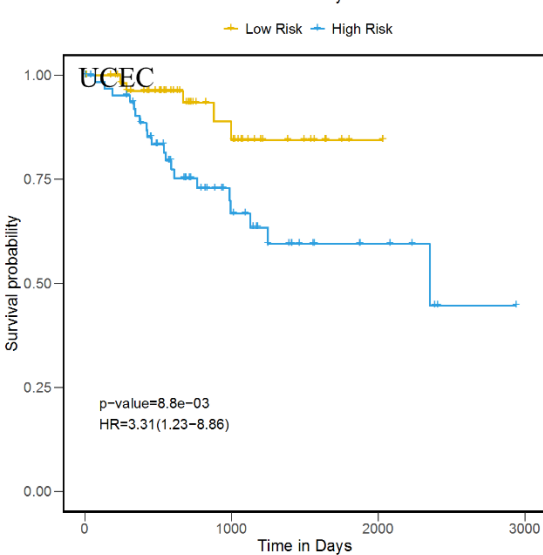

Supplement: Supplemental Information 16 — Kaplan–Meier survival analysis of the patients in each of the five cancers. The patients were divided into low-risk and high-risk groups using the median cutoff value of the partial hazard. p-value were calculated by the log-rank test [file peerj-08-9654-s016.pdf]
