# Supplementary material for: Identification of DNA methylation patterns and biomarkers for clear-cell renal cell carcinoma by multi-omics data analysis
Source: PeerJ. 2020 Aug 3;8:e9654. doi: 10.7717/peerj.9654 (PMC7409785; doi:10.7717/peerj.9654)
Supplement: Supplemental Information 13 — ROC curve for the validation datasets of GSE (BRCA), GSE (COAD), GSE (LIHC), GSE (LUAD) and GSE (PRAD). [file peerj-08-9654-s013.pdf]

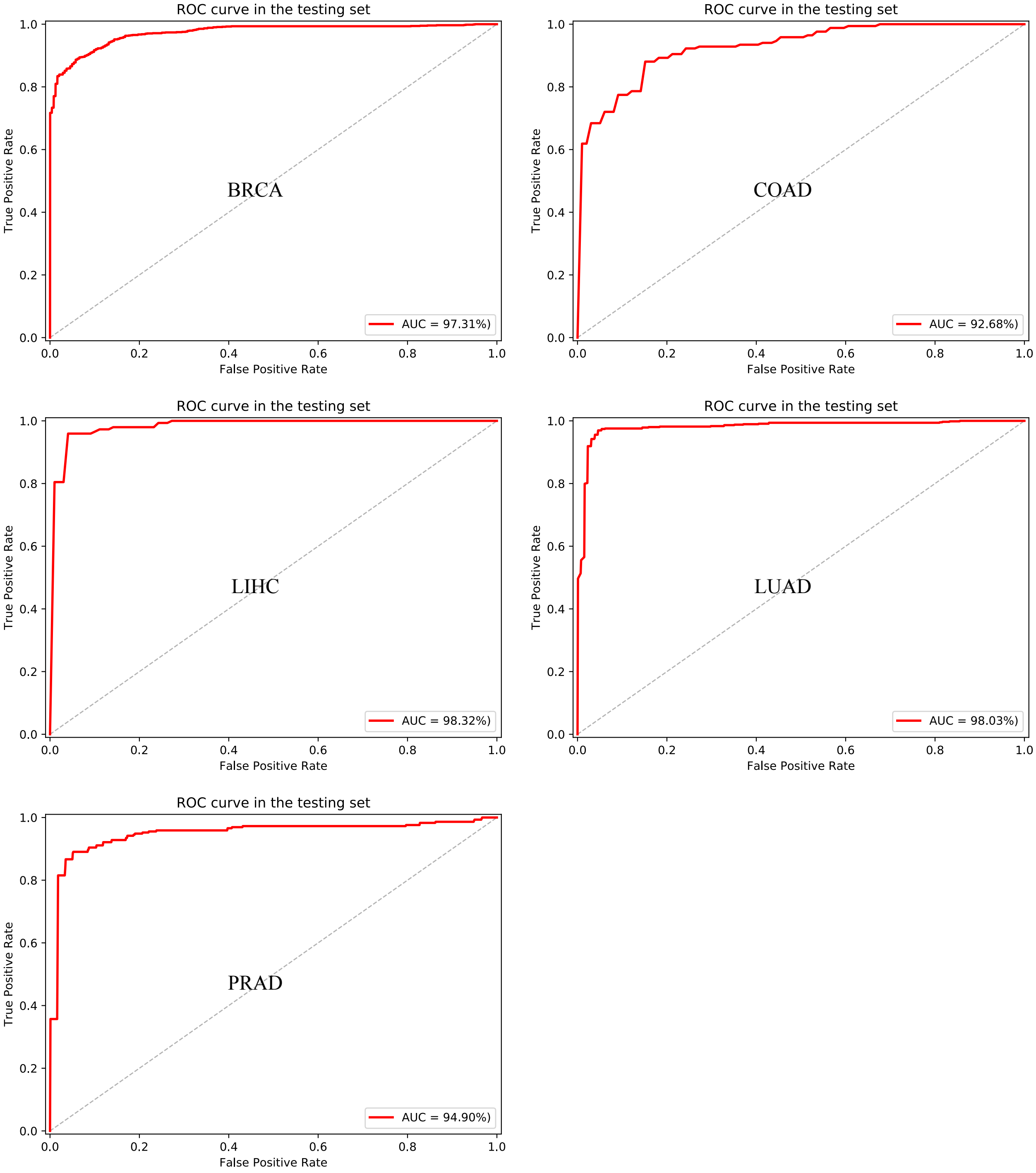
ROC curve in the testing set
BRCA
AUC = 97.31%)
ROC curve in the testing set
COAD
AUC = 92.68%)
ROC curve in the testing set
LIHC
AUC = 98.32%)
ROC curve in the testing set
LUAD
AUC = 98.03%)
ROC curve in the testing set
PRAD
AUC = 94.90%)
True Positive Rate
False Positive Rate
0.0
0.2
0.4
0.6
0.8
1.0
